# Supplementary material for: A density functional theory for ecology across scales
Source: Nat Commun. 2023 Feb 25;14:1089. doi: 10.1038/s41467-023-36628-4 (PMC9968302; doi:10.1038/s41467-023-36628-4)
Supplement: Supplementary file 1 — Supplementary Information [file 41467_2023_36628_MOESM1_ESM.pdf]

SUPPLEMENTARY INFORMATION

A density functional theory for ecology across scales

References to equations, figures, etc. refer to the main manuscript, unless indicated otherwise.

Supplementary Notes

Notes on the DFTe energy functional. By minimising the energy $E = E[\mathbf{n}, \mu](\mathbf{N})$ in equation (9), we implicitly state that the ecological system prefers realisations of lower energy. At its core, the modular decomposition of E is one of notation and not based on fundamentally disparate entities. For example, the dispersal energy in equation (2) can be thought of as an internal interaction energy, and the environmental energy can be viewed as an interaction between the system constituents and the environment. In this language we would term E a pure interaction energy. Splitting E into the parts of equation (9) merely serves the purpose of introducing and building on intuitive concepts when we determine the functional form of E. Furthermore, only relative differences to a reference energy are of consequence. For example, we may shift and/or rescale E such that its minimum equals one. Since (i) energy can be rescaled and (ii) quantities of energy and density are the only dimensionful objects that enter E, the only relevant unit in DFTe is length. In principle, the energy in equation (9) could be expressed in terms of any energy unit (e.g., Joule), but this would be (i) a tedious and ambiguous enterprise in practice and (ii) of no relevance to our analysis of ecosystems with DFTe. The parameterised components of E are not fitted to energetic data. We rather fit the minimisers of E to our only objects of interest, namely density distributions and abundances.

The potentials we introduce in equation (5) can be viewed as the Ansatz we make in DFTe. This may well prove inappropriate for certain properties of some ecosystems, but we know of no such exceptions at present. Regarding the question of existence of such potentials, we note that they cannot be measured directly. The same holds for observables in physics like electric potentials, which we have to infer by measuring the force exerted on a test charge, which itself needs to be inferred from something like a spring scale, where we read off a change of the spring's length—finally, the directly measurable quantity. Whether the electric potential 'really exists' or whether it is a mere mathematical construct is a question outside the scientific realm. However, the answer to the question of how to derive the DFTe potentials from individual-level interactions would promote the currently phenomenological DFTe to a fundamental theory.

A ubiquitous difficulty in describing many different species that interact within a complex environment is to find the proper trade-offs among given mechanisms and constraints. And this agenda gets more involved as the complexity increases. One strength of DFT-type approaches is that the complexity of the underlying functional E does not matter much as the trade-offs between the components are found automatically in the minimisation process, while the building blocks of E are parameterised independently from less complex subsystems. Although the numerical minimisation of $E[\mathbf{n}, \boldsymbol{\mu}](\mathbf{N})$ for any given system can be technically involved, the greater challenge for DFT usually lies in determining the explicit forms of the required functionals. Here, we have identified an energy functional $E[\mathbf{n}, \boldsymbol{\mu}](\mathbf{N})$ for ecology that lends itself to intuitive interpretations and quantitatively describes a wide range of ecological systems, despite its structural simplicity and with very few fitting parameters.

To once again illustrate the reasoning and simple intuition behind building DFTe energy components, we propose a model of the specific ecological phenomenon of bet hedging. This goes—specifically, not conceptually—beyond the 'major ecological principles' alluded to in the Discussion, but it does not go beyond what we proposed in equation (9). Let us suppose that a species engages in bet hedging if it gains fitness in stressful situations at the expense of fitness in favourable situations. If stressful and favourable refer to the environment (for instance, as opposed to interactions, and just to be specific here), we could perform two calculations (0) and (B), with environmental potentials $V_{\text{env}}^{(0)}$ and $V_{\text{env}}^{(\text{B})} = \left\langle V_{\text{env}}^{(0)} \right\rangle + b \left(V_{\text{env}}^{(0)} - \left\langle V_{\text{env}}^{(0)} \right\rangle \right)$ perceived by a species without (0) and with (B) bet hedging strategy, respectively. We amplify bet hedging by decreasing the parameter b from 1 to 0: We effectively decrease large (stressful) values of $V_{\text{env}}^{(0)}$ and increase small (favourable) values of $V_{\text{env}}^{(0)}$ towards the mean $\left\langle V_{\text{env}}^{(0)} \right\rangle$. Then, $b = 0$ represents maximal bet hedging because $V_{\text{env}}^{(\text{B})} = \left\langle V_{\text{env}}^{(0)} \right\rangle$ is constant, and the discrimination between stressful and favourable environments disappears. If, for a particular choice of b and taking into account all other constraints like resources, interactions, etc., the abundances obtained with the DFTe energy minimisation are $\hat{N}^{(\text{B})} > \hat{N}^{(0)}$, then we may declare bet hedging 'of relative magnitude $1 - b$' advantageous. This illustrates how DFTe can incorporate, in both an abstract and a simple way, potentially very specific ecological phenomena like different egg sizes in a clutch that may have evolved as a protection against environmental variability.

With a specific system at hand, we retrieve the appropriate parameter values in equation (9) from actual data. Once these values are identified and validated, we can predict the species densities

in new situations. Most importantly, we can predict the behaviour of complex systems by minimising $E[\mathbf{n},\mu](\mathbf{N})$ using the parameters obtained for less complex subsystems. We can also rule out interaction mechanisms that yield poor fits to data. That said, equation (9) is likely not the last word on predicting species distributions in ecology. But we can and do show consistency of equation (9) with experimental and field data across a variety of taxa and situations. In the course of studying specific experimental systems, we have justified all components of equation (9). While DFTe predictions based on less controlled field data have to be the final criterion for judging the universal applicability of equation (9) to ecology, here we focus on establishing the structure of DFTe from relatively unambiguous laboratory data and established synthetic data.

The DFTe framework is not falsifiable since additional energy components can be added to equation (1), but particular DFTe models created for specific ecosystems can be ruled out with confidence. This would be of little use if the models would comprise many energy functional components with many fit parameters and if new phenomena needed new functional forms. However, assuming that our case studies in Figs. 2–7 are representative, we have demonstrated that our four energy functionals are able to cover the basic ecosystem properties across scales, and that the parametric variability in these functionals allows us to distil general ecosystem properties. For example, DFTe fits the fruit flies data with a total of two parameters from three energy components, and we would obtain an inferior fit if we replaced the repulsive Coulomb-type interaction by an attractive interaction. This way, we have distilled that long-range repulsive behaviour rather than attraction is a fundamental property of fruit flies. Of course, specific DFTe models select a prediction from a continuum of possibilities, and do not necessarily answer yes–no questions.

In this manuscript, we have left many important aspects of ecosystem modelling untouched. For example, we have so far modelled systems only at fixed scales, but we speculate that the understanding of scaling relations across scales within the DFTe framework could come from modelling a fixed set of species with a fixed functional at different scales. If the fits to the data are of similar (and high enough) quality across these scales, we may argue that the mechanisms underlying the employed energy functional are responsible for the scaling relations we observe in the data.

We note that when fitting the DFTe energy to scattered or low-resolution data, the continuum between the data points (of, e.g., V^{env}) has to be interpolated, and the resulting predictions will reflect the quality of the chosen interpolation. In general, the number of free DFTe parameters increases with the sparsity of information, which is a challenge also for other mechanistic approaches.

Notes on the resource energy. The DFTe energy in equation (9) balances the cost due to dispersal, environment, and interactions with the cost E_{Res} of foregone resources. The latter are replenished in equilibrium and are usually consumed only in part due to the multiple constraints in a complex ecosystem. The rate of consumption is irrelevant since $\rho_k(\mathbf{r})$ is replenished at the same rate in equilibrium. For example, a 50-hectare pasture that grows biomass at 20 kg/(ha day) sustains an equilibrium abundance of 50 cows (each requiring 20 kg of grass daily), such that we may choose $\nu = 20$ and $\rho = 1000$ in equation (7). Expressing E_{Res} as the sum of

$$E_{\mathrm{dis,res}} = \int_A (\mathrm{d}\mathbf{r}) \sum_{k=1}^{K} w_k \sum_{s=1}^{S} \nu_{ks}^2 n_s^2, \tag{1}$$

$$E_{\mathrm{env,res}} = \int_A (\mathrm{d}\mathbf{r}) \sum_{k=1}^{K} w_k \left[\rho_k^2 - 2\rho_k \sum_{s=1}^{S} \nu_{ks} n_s\right], \tag{2}$$

and

$$E_{\mathrm{int,res}} = 2 \int_A (\mathrm{d}\mathbf{r}) \sum_{k=1}^{K} w_k \sum_{\substack{s,s'=1 \\ s'>s}}^{S} \nu_{ks}\, \nu_{ks'}\, n_s\, n_{s'}, \tag{3}$$

we reveal the rich interpretations of the simple expression in equation (7). $E_{\mathrm{dis,res}}$ encodes the intra-species pressure in consuming resources, akin to the dispersal energy in equation (2). The negative term $-2\sum_{k=1}^{K} w_k \nu_{ks} \rho_k$ in $E_{\mathrm{env,res}}$ modifies the environment V_s^{env} favourably and balances against the positive $E_{\mathrm{dis,res}}$ and $E_{\mathrm{int,res}}$. The constant offset produced by ρ_k^2 is irrelevant to the minimiser of E (prior to the addition of other energy components of equation (9)). $E_{\mathrm{int,res}}$ presents inter-species pressure mediated by resources (just like $E_{\mathrm{dis,res}}$ does for conspecifics) and comes in the form of a repulsion symmetric in s and s', see Supplementary Table 1. It is a special case of the more general interaction energy

$$E_{\mathrm{c}} = \sum_{\substack{s,s'=1 \\ s'\neq s}}^{S} \int_A (\mathrm{d}\mathbf{r})\, n_s(\mathbf{r})^{\alpha_s}\, \Gamma_{ss'}(\mathbf{r})\, n_{s'}(\mathbf{r})^{\beta_{s'}}, \tag{4}$$

which is itself a special case of equation (6) with $\gamma_{ss'}(\mathbf{r},\mathbf{r}') = \delta(\mathbf{r}-\mathbf{r}')\,\Gamma_{ss'}(\mathbf{r})$. In addition to the resource-based pressure, internal pressure may come from other intra-species mechanisms, such as competition for mates or territory, and can be encoded in τ_s, which lets the equilibrium value of $\sum_{s=1}^{S} \nu_{ks} n_s$ in equation (7) deviate from its a priori equilibrium value ρ_k.

intra-specific interaction	τ_s	interpretation
spreading/dispersal	> 0	avoidance of conspecifics
aggregation	< 0	beneficial proximity of conspecifics

environment	V_s^{env}	interpretation
attraction	small	favourable region (a type of niche)
repulsion	large	hostile region

bipartite interactions	$\gamma_{ss'}$	$(\alpha_s,\beta_{s'})$	impact on (s, s')
amensalism	> 0	(0,1)	(0,-)
repulsion	> 0	(1,1)	(-,-)
asymmetric interaction (e.g., parasitism)	> 0	(1,2)	(+,-)
commensalism	< 0	(0,1)	(0,+)
mutualism	< 0	(1,1)	(+,+)

Supplementary Table 1. **Links between ecological concepts/phenomena and DFTe parameters.** We summarise and complement the interpretation of the DFTe parameters introduced through equations (2), (3), and (6). All basic interactions in ecology are covered with the generalised competition energy E_γ in equation (6), which favours (+), penalises (-), or leaves the species unaffected (0). A competitive advantage of one species s over another species s' requires lower contribution to the energy if $n_s > n_{s'}$. Indeed, for $\gamma_{ss'} = \gamma_{s's} > 0$ and $\beta > \alpha$, we find s favoured over s' if $n_s^\alpha \gamma_{ss'} n_{s'}^\beta < n_{s'}^\alpha \gamma_{ss'} n_s^\beta$, which is equivalent to $n_s^{\beta-\alpha} > n_{s'}^{\beta-\alpha}$. That is, the asymmetric competition incurs a cost for the superior species, but a larger cost for the inferior species. For $\alpha = \beta$, the interaction is symmetric in s and s', such that an overlap of n_s and $n_{s'}$ is energetically favoured (punished) for $\gamma_{ss'} < 0$ $(\gamma_{ss'} > 0)$. For amensalism and parasitism, we may, for example, specify a generic coefficient such as $\gamma_{ss'} = [f_s/f_{s'} - 1]_+ \geq 0$, with species-specific fitness proxies $\mathbf{f}$.

Notes on the interaction energy. Equation (6) represents a large family of functionals, namely all possible bipartite interactions. In fact, sums of such functionals implement the more general form $\sum_{s,s'=1}^{S} \int_A (\mathrm{d}\mathbf{r})\,(\mathrm{d}\mathbf{r}')\, f(n_s(\mathbf{r}))\,\gamma_{ss'}(\mathbf{r},\mathbf{r}')\, g(n_{s'}(\mathbf{r}'))$ of equation (6) through series expansions of the functions f and g. We impose no constraints on the interaction kernel γ within the general DFTe framework, but, of course, not all of these possible functionals can be realised or could even be endowed with biological meaning.

Equation (5) requires the first functional derivative $V_i^{\gamma}[\mathbf{n}](\mathbf{r}) = \frac{\delta E_\gamma[\mathbf{n}]}{\delta n_i(\mathbf{r})}$ of $E_\gamma[\mathbf{n}]$, whose total variation is

$$\delta E_\gamma[\mathbf{n}] = \sum_{i=1}^{S} \int_A (\mathrm{d}\mathbf{r}) \frac{\delta E_\gamma[\mathbf{n}]}{\delta n_i(\mathbf{r})} \delta n_i(\mathbf{r}) \tag{5}$$

by definition and reads

$$\delta E_\gamma[\mathbf{n}] = \sum_{i=1}^{S} \Big(E_\gamma[\ldots, n_{i-1}, n_i + \delta n_i, n_{i+1}, \ldots] - E_\gamma[\mathbf{n}] \Big)_{O(\delta n_i)} \tag{6}$$

$$= \int_A (\mathrm{d}\mathbf{r})(\mathrm{d}\mathbf{r}') \left(\sum_{i,s,s'=1}^{S} \left\{ \alpha_s n_s(\mathbf{r})^{\alpha_s - 1} \delta_{is}\, \gamma_{ss'}(\mathbf{r},\mathbf{r}') n_{s'}(\mathbf{r}')^{\beta_{s'}} \delta n_s(\mathbf{r}) \right\} + \sum_{i,s,s'=1}^{S} \left\{ \beta_{s'} n_s(\mathbf{r})^{\alpha_s} \delta_{is'}\, \gamma_{ss'}(\mathbf{r},\mathbf{r}') n_{s'}(\mathbf{r}')^{\beta_{s'}-1} \delta n_{s'}(\mathbf{r}') \right\} \right) \tag{7}$$

$$= \sum_{i=1}^{S} \int_A (\mathrm{d}\mathbf{r}) \int_A (\mathrm{d}\mathbf{r}') \sum_{s=1}^{S} \Big\{ \Big[\alpha_i n_i(\mathbf{r})^{\alpha_i - 1} \gamma_{is}(\mathbf{r},\mathbf{r}') n_s(\mathbf{r}')^{\beta_s} \Big] + \Big[\alpha \leftrightarrow \beta \;\&\; \gamma_{is}(\mathbf{r},\mathbf{r}') \leftrightarrow \gamma_{si}(\mathbf{r}',\mathbf{r}) \Big] \Big\} \delta n_i(\mathbf{r}). \tag{8}$$

In going from equation (6) to equation (7), we used equation (6) with $\gamma_{ss} = 0$ and $(x+\epsilon)^\alpha = x^\alpha + \alpha\, x^{\alpha-1}\epsilon + O\left(\epsilon^2\right)$. Using equation (5), we identify $V_i^{\gamma}[\mathbf{n}](\mathbf{r})$ in equation (8), which we obtained by swapping the summation indices s and s' (integration variables $\mathbf{r}$ and $\mathbf{r}'$) in the first (second) line of equation (7). As a consistency check and for completeness we now use equation (8) to show that

$$\frac{\delta E_\gamma[\mathbf{n}]}{\delta n_i(\mathbf{r}) \delta n_k(\mathbf{z})} = \frac{\delta E_\gamma[\mathbf{n}]}{\delta n_k(\mathbf{z}) \delta n_i(\mathbf{r})}. \tag{9}$$

The parametric dependence of $V_i^{\gamma}[\mathbf{n}](\mathbf{r})$ on $\mathbf{r}$ appears both in n_i and in γ. It is therefore convenient to calculate the second functional derivative $\frac{\delta V_k^{\gamma}[\mathbf{n}](\mathbf{z})}{\delta n_i(\mathbf{r})}$ of $E_\gamma[\mathbf{n}]$ in analogy to equations (6)–(8) by

rewriting $V_k^\gamma[\mathbf{n}](\mathbf{z})$ as

$$V_k^\gamma[\mathbf{n}](\mathbf{z}) = \int_A (\mathrm{d}\mathbf{r})\,(\mathrm{d}\mathbf{r}')\sum_{s,s'=1}^{S}\left\{n_s(\mathbf{r})^{\alpha_s-1}\,A_{ss'}^k(\mathbf{r},\mathbf{r}')\,n_{s'}(\mathbf{r}')^{\beta_{s'}} + n_s(\mathbf{r})^{\alpha_s}\,B_{ss'}^k(\mathbf{r},\mathbf{r}')\,n_{s'}(\mathbf{r}')^{\beta_{s'}-1}\right\} \tag{10}$$

with $A_{ss'}^k(\mathbf{r},\mathbf{r}') = \delta_{ks}\,\delta(\mathbf{z}-\mathbf{r})\,\alpha_s\,\gamma_{ss'}(\mathbf{r},\mathbf{r}')$ and $B_{ss'}^k(\mathbf{r},\mathbf{r}') = \delta_{ks'}\,\delta(\mathbf{z}-\mathbf{r}')\beta_{s'}\,\gamma_{ss'}(\mathbf{r},\mathbf{r}')$. Then, it is straightforward to obtain the total variation of $V_k^\gamma[\mathbf{n}](\mathbf{z})$ as

$$\delta V_k^\gamma[\mathbf{n}](\mathbf{z}) = \sum_{i=1}^{S}\int_A (\mathrm{d}\mathbf{r})\frac{\delta V_k^\gamma[\mathbf{n}](\mathbf{z})}{\delta n_i(\mathbf{r})}\delta n_i(\mathbf{r}) \tag{11}$$

$$= \sum_{i=1}^{S}\int_A (\mathrm{d}\mathbf{r})\sum_{s=1}^{S}\int_A (\mathrm{d}\mathbf{r}')\Big\{\Big[f_{kis}^{\alpha\beta}(\mathbf{z},\mathbf{r},\mathbf{r}') + g_{kis}^{\alpha\beta}(\mathbf{z},\mathbf{r},\mathbf{r}')\Big] + \Big[\alpha\leftrightarrow\beta\ \&\ \gamma_{is}(\mathbf{a},\mathbf{b})\leftrightarrow\gamma_{si}(\mathbf{b},\mathbf{a})\Big]\Big\}\,\delta n_i(\mathbf{r}), \tag{12}$$

where

$$f_{kis}^{\alpha\beta}(\mathbf{z},\mathbf{r},\mathbf{r}') = \delta_{ki}\,\delta(\mathbf{z}-\mathbf{r})\,\alpha_i\,(\alpha_i-1)\,n_i(\mathbf{z})^{\alpha_i-2}\,\gamma_{is}(\mathbf{z},\mathbf{r}')\,n_s(\mathbf{r}')^{\beta_s}, \tag{13}$$

$$g_{kis}^{\alpha\beta}(\mathbf{z},\mathbf{r},\mathbf{r}') = \delta_{ks}\,\delta(\mathbf{z}-\mathbf{r}')\,\alpha_i\beta_s\,n_i(\mathbf{r})^{\alpha_i-1}\,\gamma_{is}(\mathbf{r},\mathbf{r}')\,n_s(\mathbf{r}')^{\beta_s-1}. \tag{14}$$

Both $f_{kis}^{\alpha\beta}(\mathbf{z},\mathbf{r},\mathbf{r}')$, which is proportional to $\delta_{ki}\,\delta(\mathbf{z}-\mathbf{r})$, and

$$\sum_{s=1}^{S}\int_A (\mathrm{d}\mathbf{r}')\Big\{\Big[g_{kis}^{\alpha\beta}(\mathbf{z},\mathbf{r},\mathbf{r}')\Big] + \Big[\alpha\leftrightarrow\beta\ \&\ \gamma_{is}(\mathbf{a},\mathbf{b})\leftrightarrow\gamma_{si}(\mathbf{b},\mathbf{a})\Big]\Big\}$$

$$= \alpha_i\beta_k\,n_i(\mathbf{z})^{\alpha_i-1}\,\gamma_{ik}(\mathbf{r},\mathbf{z})\,n_k(\mathbf{z})^{\beta_k-1} + \beta_i\alpha_k\,n_i(\mathbf{z})^{\beta_i-1}\,\gamma_{ki}(\mathbf{z},\mathbf{r})\,n_k(\mathbf{z})^{\alpha_k-1} \tag{15}$$

are invariant under the combined swap $[i\leftrightarrow k\ \&\ \mathbf{r}\leftrightarrow\mathbf{z}]$. Hence, the second functional derivatives of $E_\gamma[\mathbf{n}]$ commute. Of course, this also holds in the special case of $\alpha_s = \beta_s = 1$, where $\frac{\delta E_\gamma[\mathbf{n}]}{\delta n_i(\mathbf{r})\delta n_k(\mathbf{z})} = \gamma_{ik}(\mathbf{r},\mathbf{z}) + \gamma_{ki}(\mathbf{z},\mathbf{r})$, which reduces further to $\frac{\delta E_\gamma[\mathbf{n}]}{\delta n_1(\mathbf{r})\delta n_1(\mathbf{z})} = \gamma_{11}(|\mathbf{r}-\mathbf{z}|)$ if the interaction in a single-species system depends on distance alone, which is an often studied situation in physics.

Notes on an analytically solvable minimal example with resource competition. In the following, we illustrate the interplay of resources and interactions with the help of two minimal, synthetic examples. We consider uniform systems of two species that tap into resources $R_k = A\rho_k = 3$ in an area $A = 1$ and exhibit amensalism of interaction strength $\Gamma = \Gamma_{12}$, which puts species 2 ('s2') at a disadvantage relative to species 1 ('s1'), see Supplementary Table 1.

First, employing a single resource and specifying $\nu_{11} = 2$ while keeping $\nu_{12} > 0$ variable, that is, $w_1 = \frac{1}{9}\exp\left[\sigma\left(\frac{\lambda_{11}}{\lambda_{11}} - 1\right)\right] + \frac{1}{9}\exp\left[\sigma\left(\frac{\lambda_{12}}{\lambda_{12}} - 1\right)\right] = \frac{2}{9}$, we minimise the total energy

$$E(N_1, N_2) = \frac{2}{9}(2\,N_1 + \nu_{12}\,N_2 - 3)^2 + \Gamma\,N_2 \tag{16}$$

$$= \frac{2}{9}\left[\left(4\,N_1^2 + \nu_{12}^2\,N_2^2\right) + (9 - 12\,N_1 - 6\,\nu_{12}\,N_2) + (4\,\nu_{12}\,N_1\,N_2)\right] + \Gamma\,N_2 \tag{17}$$

$$= E_{\text{dis,res}} + E_{\text{env,res}} + E_{\text{int,res}} + E_c \tag{18}$$

for the admissible $N_s \geq 0$. For $\Gamma = 0$, one might expect that an increase of ν_{12}, which increases the internal pressure for s2, results in an expansion of s1. However, the dispersal energies are then exactly balanced with the increased 'resource interaction' $E_{\text{int,res}}$ between both species and the more favourable 'resource environment' $-\frac{4}{3}\,\nu_{12}\,N_2$ for s2. Indeed, the global minima of equation (16) are $E\left(N_1 = \frac{3}{2} - \frac{\nu_{12}}{2}N_2, N_2\right) = 0$ for $\Gamma = 0$, with N_2 restricted by $0 < N_2 < 3/\nu_{12}$. Coexistence of two species sustained by one resource is therefore possible for a continuum of abundances. However, for any $\Gamma > 0$ the global minimum $E\left(\frac{3}{2}, 0\right) = 0$ is unique, which means that even an infinitesimal competitive interaction settles the ambiguity in favour of the competitively superior s1.

The two species of our second toy system require two resources ($\nu_{11} = 2$, $\nu_{12} = \nu_{21} = 1$, variable $\nu_{22} > 0$). Apart from the numerical values of the parameters, the according energy functional is the one used to describe Tilman's two-species competition experiments that led us to Fig. 3. With the carrying capacities

$$(\lambda_{ks}) = \left(\frac{R_k}{\nu_{ks}}\right) = \begin{pmatrix} 3/2 & 3 \\ 3 & 3/\nu_{22} \end{pmatrix}, \tag{19}$$

the limiting resource for s1 is always R_1, while s2 is limited by R_2 (R_1) for $\nu_{22} > 1$ ($\nu_{22} < 1$). We fix the weights w_k of equation (8) by $\sigma = -4$ and then have to minimise

$$E(N_1, N_2) = w_1(2\,N_1 + N_2 - 3)^2 + w_2(N_1 + \nu_{22}\,N_2 - 3)^2 + \Gamma\,N_2. \tag{20}$$

The global minimisers $\hat{\mathbf{N}}$ of equation (20) are shown in Supplementary Fig. 1a/b, which demonstrates that already such a simple system of two species exhibits gradual suppression of either

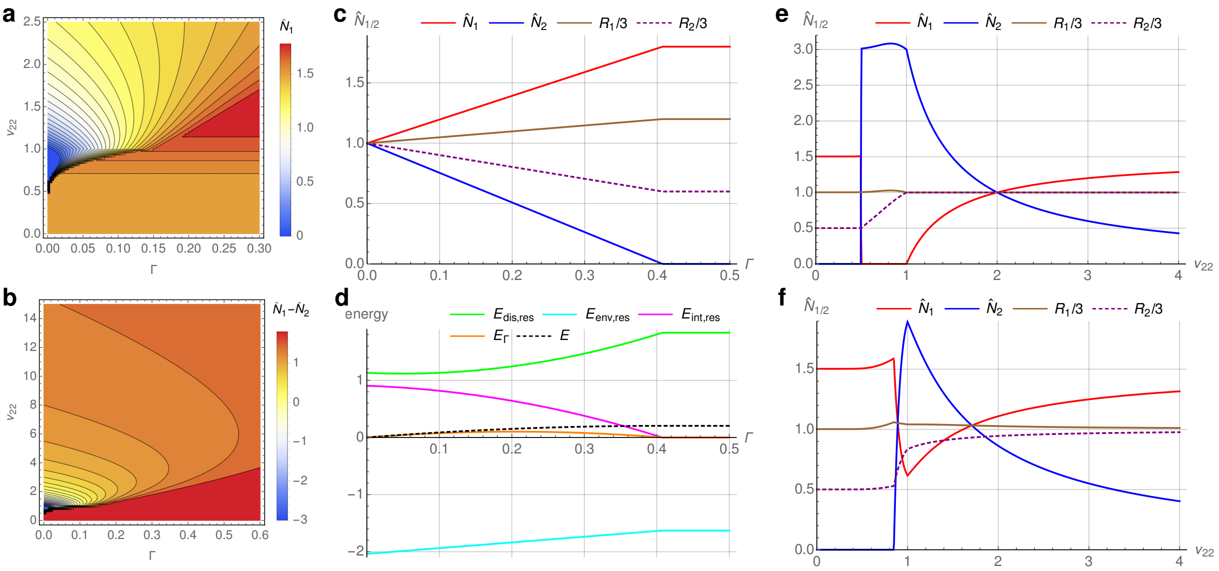

Supplementary Fig. 1. **DFTe predictions for a synthetic minimal community.** Tuning interaction strength Γ and resource requirement v_{22} of the two-species system defined through equation (20), we explore the trade-off between resource- and interaction energy, which leads to a rich variety of equilibrium states. Panel **a** shows the abundance $\hat{N}_1$ of s1, which is (generically) superior to s2, except in a small region of the parameter space where the interaction strength Γ is small enough and where the resource requirement v_{22} of s2 falls into a suitable window. The difference $\hat{N}_1 - \hat{N}_2$ in panel **b** hints at the asymptotic behaviour (i.e., competitive exclusion of s2) for large Γ and v_{22}. Panels **c** and **d** show how the departure from the symmetric situation ($\Gamma = 0$ and $v_{22} = 2$, resulting in $\hat{N}_1 = \hat{N}_2 = 1$) is accompanied by automatically determined trade-offs between the interaction energy proper E_Γ and the resource-based energy components $E_{\text{dis,res}}$, $E_{\text{env,res}}$, and $E_{\text{int,res}}$. We show the phases and phase transitions that abundances and resources undergo as v_{22} varies for constant $\Gamma = 0$ (**e**) and $\Gamma = 1/18$ (**f**).

species and competitive exclusion of one species sharply turning into competitive exclusion of the other species. Supplementary Fig. 1c depicts the gradual suppression of s2 by s1 as Γ increases at fixed $v_{22} = 2$, until s2 is competitively excluded beyond the critical value $c_{\text{crit}} = \frac{2}{5}(1 + e^{\sigma})$. As E_{Res} has to be balanced against E_Γ for $\Gamma > 0$ by permitting an energy cost that comes with foregone (R_2) and overconsumed (R_1) resources, the precise consumption of all available resources cannot be maintained. Supplementary Fig. 1d shows the energy components for the situation of Supplementary Fig. 1c. $E_{\text{int,res}}$ is largest for $\Gamma = 0$, where $N_1 = N_2$, and shrinks as the abundances increasingly deviate from the symmetric situation, until s2 is excluded. These drastic changes are driven by an amensalism energy E_c that is small throughout in comparison to the other energy components. Although N_2 vanishes beyond c_{crit}, where $E_c = 0$, amensalism is still a property of the system: s2 cannot invade if $\Gamma > \Gamma_{\text{crit}}$ since its introduction would cost too much interaction energy

E_c. This energetic condition, which emerges in the presence of amensalism during the energy minimisation, then shows up in the changed $E_{\mathrm{dis,res}}$ and $E_{\mathrm{env,res}}$ (compared with their values at $\Gamma = 0$), which add up to a finite positive value of the total energy. Supplementary Fig. 1e for the noninteracting case ($\Gamma = 0$) exhibits the basic phases of the system. Both species are on equal footing in the symmetric situation for $\nu_{22} = 2$, while $\nu_{22} > 2$ ($\nu_{22} < 2$) increases (decreases) the intra-species pressure for s2 compared to s1. Increasing ν_{22} beyond $\nu_{22} = 2$ therefore excludes s2 gradually. In a nonuniform environment an increased intra-species pressure for one species would push its density distribution into previously (too hostile) unoccupied territory, see also Supplementary Fig. 2. In a uniform environment its only effect is an increased cost due to increased intra-specific repulsion, such that the species as a whole cannot consume as many resources. The so released resources become available to other species. Conversely, a decreasing ν_{22} gradually excludes s1, until it vanishes for $\nu_{22} = 1$. For all $\nu_{22} > 1$ the two species are limited by different resources. As ν_{22} decreases below one, R_1 becomes the limiting resource also for s2 (such that $N_2 = 3$), with an increasing amount of unutilised R_2 as ν_{22} approaches $1/2$ (note that not the resource energy has to be minimised, but the total energy, such that lowering the total energy through overconsumption is permitted, which is why s2 exceeds $N_2 = 3$ for $1/2 < \nu_{22} < 1$). The cost of foregone R_2 is then so high that s1 enters the scene again—and in doing so, expels s2 completely. Already a simple system of two species (that only interact via resource consumption) thus exhibits gradual exclusion of either species ($1 < \nu_{22} < 2$ and $\nu_{22} > 2$), and competitive exclusion of s1 (for $1/2 < \nu_{22} < 1$) sharply turning into competitive exclusion of s2 (for $0 < \nu_{22} < 1/2$). Supplementary Fig. 1f for finite amensalistic interaction strength $\Gamma = 1/18$ shows the same qualitative picture as Supplementary Fig. 1e, but s2 dominates only for $0.9 \lesssim \nu_{22} \lesssim 1.7$ instead of $1/2 < \nu_{22} < 2$, and s1 is never fully excluded. Finally, we illuminate the role of σ in the weights of equation (8) by observing that $\sigma \to -\infty$ yields $(w_1, w_2) = (1/9, 1/9)$ for $\nu_{22} > 1$. Both resources are equally important, and the two species coexist with unique abundances for each value of ν_{22}. For $\nu_{22} < 1$, both species are limited by R_1, such that $(w_1, w_2) = (2/9, 0)$ reduces the system to our first example above (with $\nu_{12} = 1$). In contrast, a finite σ implies finite weights for both resources and all ν_{22}, resulting in the unique abundances shown in Supplementary Fig. 1e/f. Our conclusion for this minimal example of two species therefore is that (i) the advantage of s1 over s2 due to amensalism is an advantage for all resource requirements ν_{22}—relative to the noninteracting case—and (ii) either species can suppress the other depending on ν_{22} (or, alternatively, the provided resource combination).

Notes on Fig. 2. In Ref. [25] (see also Supplementary Ref. [1]), Méndez-Valderrama *et al.* set up a 'density-functional fluctuation theory' (DFFT) and obtain remarkably accurate density distributions of a single species (*Drosophila melanogaster*) in a heterogeneous environment (holding chambers with one side exposed to a heat source). They employ the grand-canonical ensemble of statistical physics with an energy functional whose system-specific parameters are extracted directly from the data: While they assume an environmental energy functional of the form in equation (3) to capture the preferential positions of fruit flies subjected to a temperature gradient in the chambers, the intra-specific interaction functional remains unspecified. This approach is particularly useful if nothing is known about those interactions or if no functional form should be assumed. Its applicability to more complex situations remains to be studied, though. Most importantly, resources are not considered since a fixed number of fruit flies is constrained to the chambers for a limited time. This situation represents the fundamental building block of our DFTe framework: Calculating the density distributions for fixed abundances, whatever the energy functional. In contrast to DFFT, we have to specify an interaction functional and explicitly parameterise an environment. This can be seen as a disadvantage since we impose mechanisms and structure on the ecosystem model. But it also means that we are able to discriminate between mechanisms, can dismiss those that are inconsistent with the data, and thereby gain insight that is directly interpretable.

We extract a smooth and monotonic reference density $n_{\text{ref}}^{\text{1Dc}}$ from the experimental data points from the quasi-1D chamber (with area $A = 10 \times 0.8\,\text{cm}^2$ and 24×2 bins) of Ref. [25] by averaging pairs of the 48 bins in descending order. The cubically interpolated result is shown as the red dotted line in Supplementary Fig. 2 and resembles an inverted parabola, such that the density expression in equation (4) yields a first approximation of $n_{\text{ref}}^{\text{1Dc}}$ for $V(\mathbf{r}) = V^{\text{env}}(\mathbf{r}) \propto (x + x_0)^2$ and $x_0 = 5\,\text{cm}$. We acknowledge in the Discussion that the intuition required for setting up a DFTe energy functional can be a barrier of entry for applying DFTe. Such difficulties are not unexpected when introducing a fundamentally novel approach, and we can only hope that detailed accounts of our reasoning will be adequate guides. Therefore, we offer an alternative phrasing of the reasoning behind equation (24): Equation (4) is essentially $n(x) = \text{constant} - V(x)$, and the noninteracting situation $V(x) = V^{\text{env}}(x)$ produces a first approximation (cf. $n_{\tau=1}^{(0)}$ in Supplementary Fig. 2b) of $n_{\text{ref}}^{\text{1Dc}}(x)$ if $V^{\text{env}}(x)$ is proportional to something like $x^{3/2}$, or x^2, or $x^{5/2}$, which we guess just by looking at the red dashed line in Supplementary Fig. 2b; let us take x^2. Since we implicitly assume

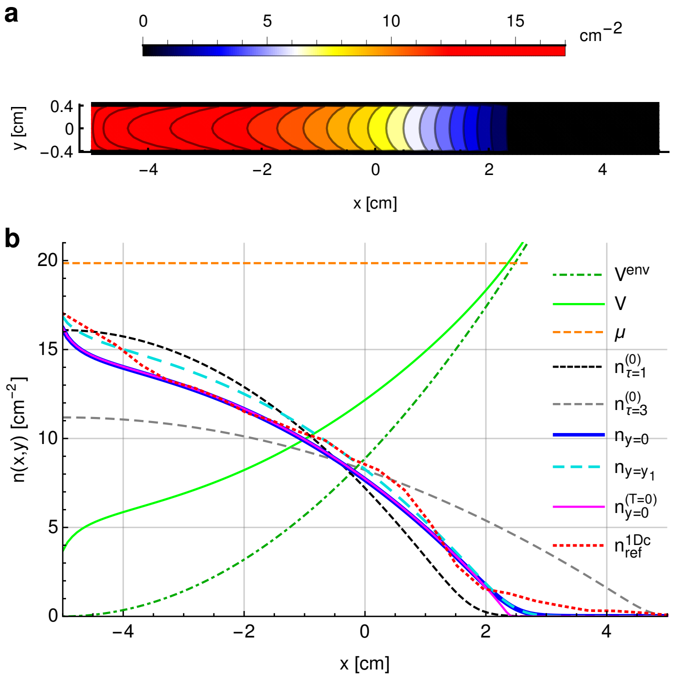

Supplementary Fig. 2. **Interpretation of the TF density from DFTe fits to fruit flies. a**, Equation (24) with $\varepsilon = 35.5\,\text{cm}^{-2}$ and $\gamma = 9\,\text{cm}$ yields the DFTe equilibrium densities of 65 fruit flies in the quasi-1D chamber ('1Dc'; area $A = 10 \times 0.8\,\text{cm}^2$; heat source at $x = 5\,\text{cm}$; from Ref. [25]) as least-squares fits to the experimental data interpolated by $n^{1\text{Dc}}_{\text{ref}}$. A priori, τ, which enters equation (24) via equation (2) is a fit parameter as well, but a rescaling of the energy does not affect its minimiser. We thus may set $\tau = 1\,\mathcal{E}\mathcal{L}^2$, in our units of energy [$\mathcal{E} = 1$] and length [$\mathcal{L} = 1\,\text{dm} = 10\,\text{cm}$], since we only seek densities and relative energy differences. **b**, The energies V, V^{env}, and μ are shown rescaled by $1/100$. With the quadratic environment in equation (24), the TF density formula in equation (4) produces an inverted parabola for the density distribution prior to interactions—a first approximation of $n^{1\text{Dc}}_{\text{ref}}$. The cuts n_y along the x-direction for $y = 0$ and $y_1 = 0.391\,\text{cm}$ give an average account of $n^{1\text{Dc}}_{\text{ref}}$. Owing to the nonlocal repulsion, the density increases towards the chamber boundaries at $y = \pm 0.4\,\text{cm}$, but this is a small effect and of no qualitative importance here: The density profiles along the x-direction are a reasonable fit to $n^{1\text{Dc}}_{\text{ref}}$ for all y. We obtain the results in Fig. 2 using equation (23) with $T = 100$, which produces a smooth version of $n^{(T=0)}_{y=0}$ (viz., equation (4)) in the vicinity of the habitable zone boundary (the analogue of the quantum-classical boundary) at $V = \mu$. In the absence of interactions in equation (24), V equals V^{env} and yields $n^{(0)}_{\tau=1}$ (for $\varepsilon = 35.5\,\text{cm}^{-2}$), which reveals the repulsive nature of both the interaction and the dispersal energy when compared with $n_{y=0}$ and $n^{(0)}_{\tau=3}$, respectively. The dispersal energy turns out to be less than 8% of the total energy. If this is not negligible, then the fruit flies engage in a contact-type repulsion alongside the finite-range repulsion, which comprises about 32% of the total energy.

that all flies are identical, the interaction between two flies located at x and x' has to be symmetric, i.e., the interaction integrand of equation (6) is $n(x)^\alpha\,\gamma(x, x')\,n(x')^\alpha$. The existence of territoriality among fruit flies tells us that the interaction kernel $\gamma(x, x')$ should be positive and should have a finite range. Hence, $\gamma(x, x')$ depends in some way on $|x - x'|$. Since we deem it natural to

assume that γ decreases with distance, the parsimonious choice is $1/|x - x'|$. Finally, we opt for the parsimonious choice $\alpha = 1$. The dispersal energy in equation (24), which facilitates execution of the selfconsistent DPFT loop, could have been rendered irrelevant if the fit parameters ε and/or γ had turned out very large (in terms of the energies they produce relative to the dispersal energy). In a sense, the data itself takes care of suppressing irrelevant energy components.

We assess the quality of predictions vs data with the least-squares correlation measure

$$\xi = \frac{2\,\mathbf{p}\cdot\mathbf{d}}{\mathbf{p}^2 + \mathbf{d}^2}, \tag{21}$$

illustrated in the table below. We also note that uniformly drawn unit vectors $\mathbf{p}$ with positive entries in three dimensions yield the expectation value $\langle\xi\rangle = 1/2$ for any given unit vector $\mathbf{d}$.

test vector vs. (100,10,1,0)	ξ
(110,10,1,0)	0.996
(100,1,0,0)	0.996
(200,10,1,0)	0.801
(200,0,0,0)	0.798
(30,30,30,30)	0.486
(0,1,10,100)	0.002

Notes on Fig. 3. We find a valuable test system for DFTe in Tilman's seminal study of resource competition (R^*-theory) among four diatoms of Lake Michigan [1]. While the experimental data of abundances in Ref. [1] are limited to two species competing over two resources (SiO_2,PO_4) for 30 days (validating the R^*-predictions reasonably well), R^*-theory is applicable to any number of species and resources over any period of time. Although both data and predictions in Fig. 6 of Ref. [1] seemingly approach equilibrium abundances of coexisting F and A, the actual R^*-equilibrium from Tilman's fitted model (approached as the observation time tends to infinity) amounts to competitive exclusion of A and F, respectively, for two of the three employed resource combinations, see Supplementary Table 2. In fact, besides cases with involvement of the inferior T, for which both data and R^*-theory suggest exclusion of T under all circumstances, five out of the nine remaining cases are not even remotely (R^*-)equilibrated after 30 days. Since we want to test the equilibrium predictions of DFTe, we thus refrain from benchmarking against the actual (transient) data and rather aim at reproducing the equilibrium abundances that follow from R^*-theory. Strictly speaking, we are not dealing with a two-dimensional system here, but one

may simply think of the third direction integrated out. The uniform setup with local interactions allows us to set the unit of length arbitrarily, without consequences for the determination of the equilibrium abundances. We set $A = 1$, which amounts to 1 mL suspension by our definition. The unit of energy is then implicitly fixed via $\zeta = 1$ in equation (7).

		abundances [in 1 mL suspension]				
species	resources	experiment	predictions from R^*-theory		DFTe predictions	
	(SiO_2,PO_4)	after 30 days	after 30 days	at equilibrium	at equilibrium	ξ
(F|A)	$\mathcal{R}_1$	(6,500|3,000)	(3,880|4,805)	(11,330|0)	(12,370|0)	0.996
(F|A)	$\mathcal{R}_2$	(14,000|28,000)	(6893|9,666)	(0|22,140)	(0|22,310)	1.000
(F|A|S)	$\mathcal{R}_1$	—		(11,330|0|0)	(12,370|0|0)	0.996
(F|A|S)	$\mathcal{R}_2$	—		(0|21,940|46)	(0|21,660|152)	1.000
(F|A|S)	$\mathcal{R}_3$	—		(0|0|1,801)	(0|0|1,818)	1.000
(F|A|S)	$\mathcal{R}_4$	—		(0|4,850|4,086)	(0|4,564|4,193)	0.999
(F|A|S)	$\mathcal{R}_5$	—		(375,872|89,578|0)	(349,715|109,230|0)	0.996
(F|A|S)	$\mathcal{R}_6$	—		(0|350|64)	(0|71|170)	0.447
(F|A|S)	$\mathcal{R}_7$	—		(113,567|96,250|0)	(108,983|100,443|0)	0.999
(F|A|S)	(350,6.05)	—		(0|232,500|0)	(0|232,300|30)	1.000
(F|A|S)	(56.2,0.14)	—		(0|1,288|936)	(0|1,567|830)	0.984

Supplementary Table 2. **Analysis of R^*-predictions for benchmarking DFTe.** The (F|A) subset of the four species (F|A|S|T) studied in Ref. [1] suggests to benchmark the DFTe trade-off between resources and competition against R^*-equilibria instead of the nonequilibrated experimental abundances after 30 days. Furthermore, the resource combinations $\mathcal{R}_1 = (12, 10)$, $\mathcal{R}_2 = (41.3, 0.58)$, and $\mathcal{R}_3 = (209, 0.2)$ in units of μmol/L, used for the experiments in Ref. [1] do not lead to strong signatures of coexistence: Competitive exclusion prevails apart from the two borderline cases $(F|S)|_{\mathcal{R}_2} = (11, 240|425)$ and $(A|S)|_{\mathcal{R}_2} = (21, 940|46)$, where S is almost excluded by F and A, respectively. We cover a broader range of abundances by considering four additional resource combinations ($\mathcal{R}_4 = (250, 0.58)$, $\mathcal{R}_5 = (500, 20)$, $\mathcal{R}_6 = (9.991, 0.0206)$, and the average $\mathcal{R}_7 = (255.6, 7.845)$ of $\mathcal{R}_{1\text{–}6}$) that result in indisputable coexistence according to R^*-theory. In all our calculations, DFTe never predicts coexistence of three species, in line with R^*-theory. Since T is always excluded, the most interesting assemblage is (F|A|S), then equivalent to (F|A|S|T) at equilibrium, where each species is able to competitively exclude the others depending on the provided resources. DFTe also shows that A can coexist with either F or S, while F and S cannot coexist—in agreement with R^*-theory. Our results match the R^*-predictions quantitatively. The bottom row for (F|A|S) details the case of smallest overlap ξ between DFTe- and R^*-equilibria found among all 500 of our randomly chosen resource combinations, see Fig. 3. The observed discrepancies between DFTe and R^*-theory are most visible when the resource combinations are very close to the R^*-values of the involved algae (e.g., $\mathcal{R}_6$ for (F|A|S)). These disparities are accounted for by the fact that E_{Res} permits complete resource consumption (see Supplementary Notes).

The suspension for the diatoms represents a uniform environment with nutrient medium. That is, we may choose $V_s^{\text{env}} = 0$ for all species s, and consider the resource energy as part of the total energy. We disregard dispersal energy until we need it—our simulations eventually suggest that we do not. Hence, $E(\mathbf{N}) = E_{\text{Res}}(\mathbf{N}) + E_\gamma(\mathbf{N})$. Since Tilman's experiments deal with competitive exclusion, we consider $E_\gamma(\mathbf{N})$ to represent amensalism and, alternatively, what we term asymmetric competition in Supplementary Table 1. Both interactions assign a competitive advantage of one species over another, in contrast to the symmetric repulsion of type $N_s\gamma_{ss'}N_{s'}$. We can settle for amensalism only after our simulations with the asymmetric competition yield results that contradict the data. First, however, we have to specify the interaction kernels $\gamma_{ss'}$, which we use for both amensalism and asymmetric competition, see equation (12). In the setup of Ref. [1] the algae are primarily characterised through the R^*-values

$$(R^*_{sk}) = \begin{pmatrix} 1.0 & 0.005 \\ 1.0 & 0.004 \\ 5.7 & 0.002 \\ 9.7 & 0.02 \end{pmatrix} [\mu\text{mol/L}] \tag{22}$$

and nutrient requirements

$$(\nu_{ks}) = \begin{pmatrix} 9.7\times10^{-7} & 1.5\times10^{-6} & 5.8\times10^{-5} & 6.3\times10^{-6} \\ 4.7\times10^{-8} & 2.6\times10^{-8} & 1.1\times10^{-7} & 1.9\times10^{-7} \end{pmatrix} [\mu\text{mol/cell}] \tag{23}$$

that are taken from Table 1 of Ref. [1] and enter equation (28). Lacking other options, we construct fitness proxies from these two quantities. We find it plausible that smaller R^*-values and smaller nutrient requirements could both indicate higher fitness. That is, one parsimonious choice of fitness proxies are the inverses of these values, or rather the sum of inverses over the $K = 2$ resources. We thus arrive at the two potentially influential fitness proxies f_s and g_s of equations (26) and (27) by including the weights w_k, which acknowledge that the relative importance of resources varies depending on the supplied resource combination. We may then postulate the generic interaction kernels $\gamma_f\,[f_s/f_{s'} - 1]_+$ and $\gamma_g\,[g_s/g_{s'} - 1]_+$ according to Supplementary Table 1, with two fit parameters γ_f and γ_g that imply two interaction energies of the type in equation (12). The magnitudes of γ_f and γ_g inform us about the absolute and relative importance of both types of fitness proxies and their associated interaction energies. Alternatively, we can set up the single interaction kernel in equation (25), where the relative importance of both types of fitness proxies is cast into the exponent κ (where $\kappa \gg 1$ renders the fitness proxies g_s irrelevant), and where γ encodes the

importance of the interaction energy relative to (and thus enables the trade-off with) the resource energy E_{Res}. The only free parameter in E_{Res} is σ. Our choice $\sigma \to -\infty$ makes nonlimiting resources irrelevant. Finally, we note that the energy function $E(\mathbf{N})$ in equation (28) requires input from monoculture data only.

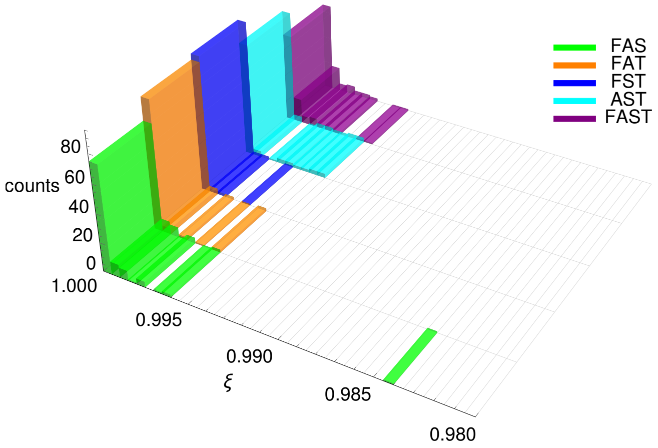

Supplementary Fig. 3. **Summarising histogram of the results shown in Fig. 3.** Comparing the DFTe predictions with the R^*-predictions for randomly chosen resource combinations [100 cases each for (F|A|S), (F|A|T), (F|S|T), (A|S|T), (F|A|S|T)] in the ranges [9.991 . . . 500] for SiO_2 and [0.0206 . . . 20] for PO_4, we find a near-perfect match in all cases. The lower limits of the resource ranges are 3% above the maximal R^*-values (9.7 SiO_2 and 0.02 PO_4 for T). The smallest overlap ($\xi \approx 0.984$) between DFTe and R^*-theory among all our resource cases is (incidentally) encountered for one resource case of (F|A|S), see Supplementary Table 2.

The two free parameters of $E(\mathbf{N})$ (γ and κ in the interaction term) are fitted exclusively to all two-species subsets of (F|A|S|T) subjected to the reference resource combinations $\mathcal{R}_{1-7}$, with an average of $\xi \approx 0.992$ for these 42 cases. The best fit ($\gamma = 8 \times 10^{-8}$ and $\kappa = 8$) maximises ξ among all those parameters that yield the correct survivors (according to R^*-theory). Supplementary Fig. 3 shows a histogram for 500 randomly drawn resource combinations that yield correlations of $\xi \approx 1$ between DFTe and R^*-theory, as discussed in Fig. 3. Complete correlation ($\xi = 1$) is not expected since the generic resource energy of DFTe is void of species-dependent threshold resource densities R^*, which are corner stones of Tilman's resource competition theory, and thus permits complete consumption of all provided resources. Accordingly, small values of ξ (like for (F|A|S) subjected to $\mathcal{R}_6$, see Supplementary Table 2) are encountered for resource combinations close to the R^*-values. We emphasise, however, that the quantitative differences between DFTe and R^*-theory in such cases is largely accounted for by DFTe's (over-)consumption of magnitude R^*: If we artifi-

cially reduce the R^*-values by a factor of 1000, R^*-theory indeed predicts (F|A|S) = (0|72|170) for $\mathcal{R}_6$, which is almost exactly the DFTe prediction in Supplementary Table 2. Note that we reduce the R^*-values only here, and for the sole purpose of demonstrating that our choice of the DFTe energy in equation (28) for Tilman's experiment is appropriate if the supplied resources are not too close to the R^*-values. We used the unchanged R^*-values from Ref. [1] for all outcomes presented in Results and in Supplementary Table 2. A corrective energy term that captures each species survival thresholds of resources densities $\rho^*_{sk} = R^*_{sk}/A$ can be introduced, for example, through a species-specific environmental potential

$$V_s^{\text{env}}(\mathbf{r}) = \sum_{k=1}^{K} \exp\left[\chi\left(\rho^*_{sk} - \rho_k(\mathbf{r})\right)\right] \tag{24}$$

which exponentially penalises species s at $\mathbf{r}$ if any $\rho_k(\mathbf{r})$ approaches or falls below ρ^*_{sk}. A priori, χ is an additional (likely large positive) fit parameter.

With $\kappa = 8$, the fitness proxies g_s based on resource requirements per cell are virtually irrelevant compared to the fitness proxies f_s that feature the R^*-values. They are not completely superfluous, though, since F and A both exhibit $R^* = 1$ under SiO_2 limitation, which would render both species noninteracting [in the sense of equation (25)] if it were not for their differing resource requirements. Of course, this is a fine-tuning artefact of numerically identical R^*-values—that is, equation (25) is likely independent of ν in realistic communities.

Notes on Fig. 4. The purpose of this example is two-fold. Our first goal is to predict densities of species in a nonuniform environment using data of experiments carried out in a uniform setting. Second, we apply DFTe to data that come with very limited information on mechanisms and species properties—a situation that requires us to postulate a functional form of E (informed by the available data) and to fit many parameters of E.

Kenkel *et al.* in Ref. [34] report the response of three grasses [*Poa pratensis* (Poa), *Hordeum jubatum* (Hord), and *Puccinellia nuttalliana* (Pucc)] to eight salinity levels. All three plants perform best at lowest salinity when grown in monoculture, but competitive interactions change this pattern when grown in mixture, see Supplementary Fig. 5a, where we replicate the data of Ref. [34] for the above-ground biomass in grams, collected after 94 days of growth from pots of 10 cm diameter (corresponding to $A = 1$ in our units) that were seeded with 150 plants per species.

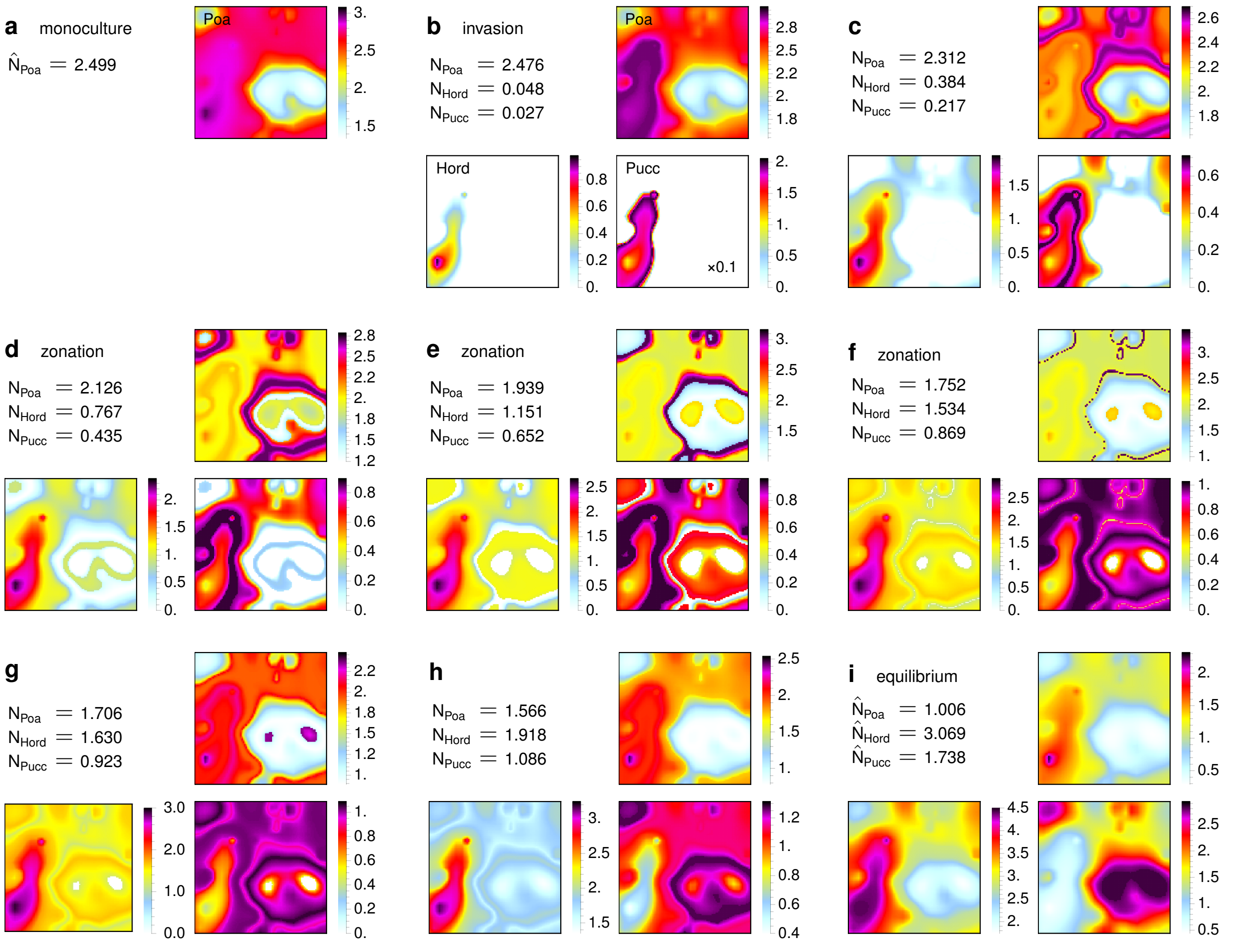

Supplementary Fig. 4. **Three grasses on a trajectory to equilibrium.** The density distributions of Poa, Hord, and Pucc exhibit a zoo of phases that emerge, transform, and disappear as we trace out a straight line in **N**-space, from Poa's monoculture distribution (**a**) to the fully equilibrated mixture of the three species (**i**). In view of the very complex trade-offs between the mechanisms and constraints, the following narrative should be regarded as a plausible interpretation of the qualitative features of this sequence rather than a rigorous account. Hord and Pucc start to invade in areas of highest resource densities (**b**). The competitively superior Hord thereby pushes Pucc into less habitable regions within the high-resource area they both occupy. This effect becomes more pronounced and also impacts Poa visibly as N_{Hord} and N_{Pucc} increase (**c**). Sharp vegetation zone boundaries start to appear and intensify for all species (**d** and **e**). The narrow high-density niches where Poa aggregates in **e** are almost vanquished in **f**, with Hord and Pucc filling the gap. Somewhat counterintuitively, two enclaves in the bottom right quadrant permit high density of Poa in **g**, despite the low resource concentration. Evidently, the minimisation of the total energy favours letting a part of Poa's fixed (excessive) abundance $N_{\mathrm{Poa}} = 1.706 (> \hat{N}_{\mathrm{Poa}} = 1.006)$ aggregate in a less preferable habitat as opposed to Poa bearing the cost of competition with Hord and Pucc in the high-resource areas. These two refuges disappear in **h** due to (i) increased competition with Hord and Pucc, and (ii) smaller N_{Poa}. Overall, however, **g** already exhibits the main features of the final (smooth) equilibrium in **i**, with **h** depicting an intermediate stage.

All experiments were performed in triplets, with averages presented in Supplementary Fig. 5a for each of the eight NaCl concentrations from 0 to 14 g/L and for each species in both monoculture and mixture. The salinity level is constant in each experimental pot, such that the data points in Supplementary Fig. 5a are to be viewed as isolated. We assume that the data represent equilibria.

The final abundances are bounded from above by the constant concentration of the nutrient medium (which includes the salt, but remains unspecified otherwise) and accessible pot area. Without loss of generality, resource R_s limits species s with $\nu_{ss} = 1$, such that the monoculture abundance N_s in Supplementary Fig. 5a equals the provided amount of resource R_s (for $\tau_s = 0$). These limiting resources also put upper limits on the six off-diagonal resource requirements ν_{ks} with $k \neq s$. We use an interaction strength parameter γ factored into $\gamma_{ss'} = \gamma\,[f_s/f_{s'} - 1]_+$ and the same fitness proxies f_s for all three types of competitive bipartite interactions (amensalism, repulsion, and asymmetric interactions, see Supplementary Table 1). As Fig. 4 of Ref. [34] suggests, we fix the salinity($\mathcal{S}$)-dependent fitness proxies f_s as the fraction of above-ground biomass in mixture, approximated by the shifted Gaussians

$$f_{\mathrm{Poa}} = 0.670\,\exp\left(-0.0030\,(\mathcal{S} + 15)^2\right) \tag{25}$$

$$f_{\mathrm{Hord}} = 0.525\,\exp\left(-0.0026\,(\mathcal{S} - 5)^2\right) \tag{26}$$

$$f_{\mathrm{Pucc}} = 0.530\,\exp\left(-0.0022\,(\mathcal{S} - 22)^2\right). \tag{27}$$

The dispersal encodes an intra-species repulsive interaction, which supplements the inter-species interactions. Finally, the free parameter σ in equation (8) allows us to fit the weights w_k of the three resources. We employ the units of length and energy, implicitly defined by $A = 1$ in equation (29) and $\zeta = 1$ for the resource energy in equation (7), respectively.

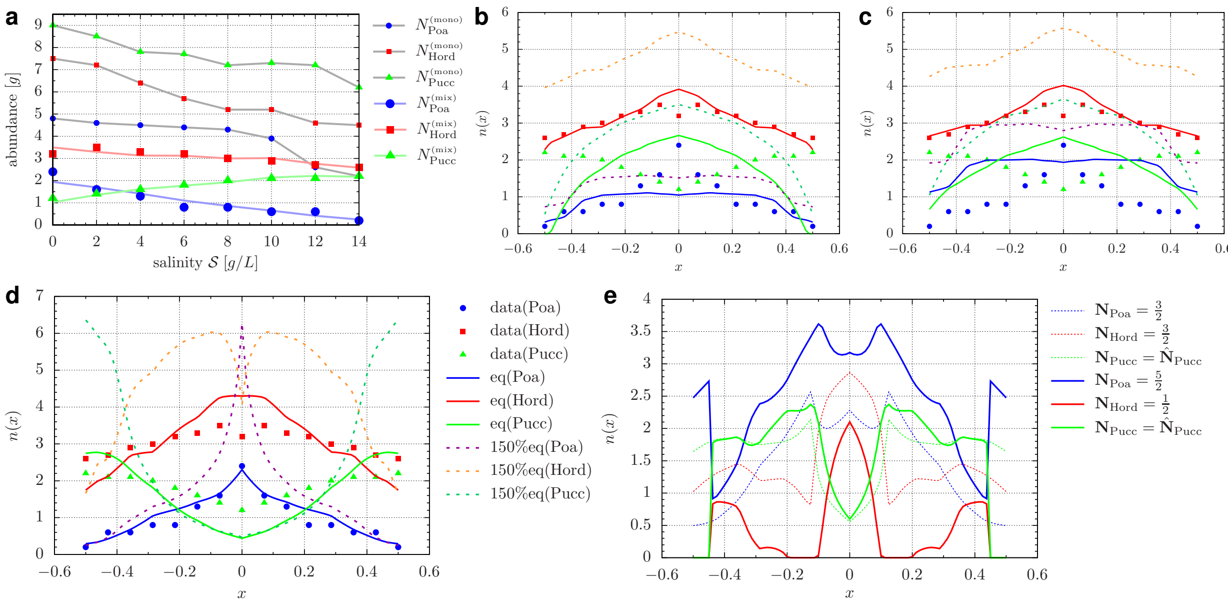

Supplementary Fig. 5. **Fits to uniform salinity inform DFTe predictions for a salinity gradient. a**, The experimentally observed equilibrium abundances (in grams) of Poa, Hord, and Pucc as a function of (constant) NaCl concentrations (in grams per litre). Salinity-dependent competition of some kind is at work since the mixture abundances $N^{(\text{mix})}$ are not simply rescaled monoculture abundances $N^{(\text{mono})}$. The grey lines through the monoculture data guide the eye. The colour lines follow from the best fits of the DFTe function in equation (29) with asymmetric interaction to the mixture abundances, see Supplementary Fig. 6. The fits for amensalism and repulsion are of similar quality. We use the resulting fitted DFTe parameters to predict the equilibrium density profiles from minimising the nonuniform version of equation (29) for a 'V'-shaped linear salinity distribution $\mathcal{S}(x, y) = 28\,|x|$ (constant in y-direction). The salinity $\mathcal{S}$ is mapped to the values of the resources R_1, R_2, and R_3 by means of the functional relationship between $\mathcal{S}$ and the monoculture abundances of panel **a**. Obviously, the three resources are highly correlated. Panels **b** and **c** for amensalism and repulsion, respectively, show similar trends when compared with the uniform abundances: Hord is largely unaffected, Poa's distribution flattened, and Pucc inverted in response to the salinity gradient. In particular the latter outcome contrasts with field observations, where Pucc is competitively displaced to areas of high salinity levels, see Supplementary Ref. [2]. We recover the trends of these field observations when employing asymmetric interactions in panel **d**. Forcing the system into a nonequilibrium high-density state ['150%eq(·)'] by fixing the abundances at 50% above the equilibrium $\hat{\mathbf{N}}$ ['eq(·)'] , we reveal more clearly the habitat niches that each species prefers under pressure. The density profiles in panel **e** showcase a situation where Pucc is kept at its equilibrium value $\hat{N}_{\text{Pucc}} = 1.611$ of panel **d**, see Supplementary Fig. 6, while $N_{\text{Poa}} > \hat{N}_{\text{Poa}}$ and $N_{\text{Hord}} < \hat{N}_{\text{Hord}}$. We observe zonation, discontinuities, and competitive exclusion along the salinity gradient.

	(ν_{ks})	τ	γ	σ	$\hat{\mathbf{N}}$ (lin)	zonation equilibrium (lin)	zonation equilibrium (ran)	zonation out-of-eq. (lin)	zonation out-of-eq. (ran)
amensalism	$\begin{pmatrix} 1 & 0.087 & 0.166 \\ 0.002 & 1 & 0.321 \\ 0.007 & 1.159 & 1 \end{pmatrix}$	0.115	0.057	-1.006	$\begin{pmatrix} 0.906 \\ 3.115 \\ 1.740 \end{pmatrix}$	×	×	×	×
repulsion	$\begin{pmatrix} 1 & 0.113 & 0.289 \\ 0.002 & 1 & 0.047 \\ 0.001 & 1.159 & 1 \end{pmatrix}$	0.093	0.019	-2.651	$\begin{pmatrix} 1.806 \\ 3.229 \\ 1.868 \end{pmatrix}$	×	×	×	×
parasitism	$\begin{pmatrix} 1 & 0.420 & 0.348 \\ 0.001 & 1 & 0.628 \\ 0.980 & 0.947 & 1 \end{pmatrix}$	0.081	0.011	-2.806	$\begin{pmatrix} 1.096 \\ 3.138 \\ 1.611 \end{pmatrix}$	×	×	✔	✔

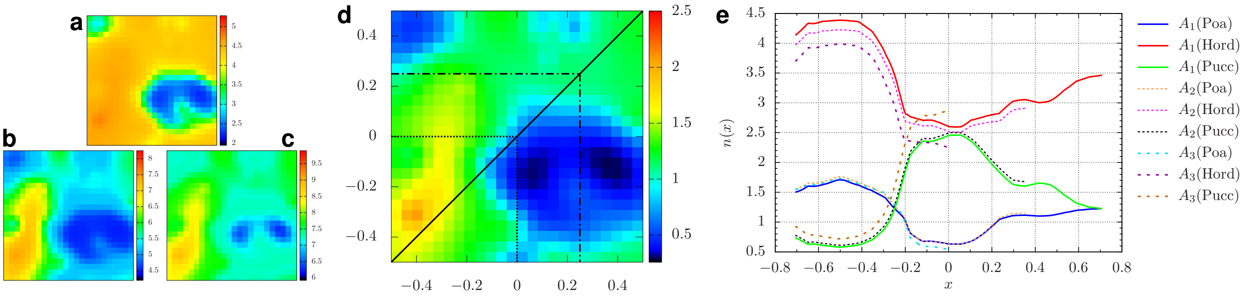

Supplementary Fig. 6. **Fitted parameters and further details on the DFTe model.** Fitting the energy function of equation (29) to the mixture abundances shown in Supplementary Fig. 5a, we obtain the six off-diagonal resources requirements ν_{ks}, the dispersal coefficient τ, the interaction strength γ, and σ for the resource weights. The three resources associated with a randomly generated salinity landscape are depicted in panels **a**–**c**. The equilibrium density distribution of Poa, depicted in panel **d**, deviates from its limiting resource R_1 (**a**) due to two mechanisms: (i) the competition with Hord and Pucc, and (ii) the intra-specific dispersal pressure that prohibits complete utilisation of the provided resources. We identify the quantitative impact of either of the two mechanisms by comparing panel **d** here with panel **a** of Supplementary Fig. 4, where Poa in isolation maximises the consumption of its limiting resource and is only restrained by the intra-specific dispersal pressure. Panel **e** shows the densities along a cut (solid diagonal line of panel **d**) through area $A_1 = A$ for all three species and reveals some quantitative (albeit no qualitative) changes when equilibrating in the restricted smaller parcels A_2 (A_3) enclosed by the dash-dotted (dashed) lines of panel **d**. The disparities between the densities for different areas reflect the altered trade-offs between the overall resource consumption and the other contributions to E, while the congruence between the density patterns reflects the local nature of the interactions and resource dependencies.

We now ask what happens if we connect the pots, such that the grasses are allowed to aggregate in their preferred habitat of low salinity at the expense of stronger competition. This question obviously emerges as we shift focus from the laboratory to realistic environments. We pursue an answer by first fitting the nine parameters of the DFTe energy functional in equation (29) to the constant-salinity data and then predict the density distributions of Poa, Hord, and Pucc in heterogeneous saline environments. We never find zonation at equilibrium, see Supplementary Fig. 5b–d and Supplementary Fig. 6, where the equilibrium density profiles turn out to be void of any discontinuities. This observation holds across the three basic types of competitive interactions and for all salinity landscapes considered, be it a linear (lin) salinity distribution or randomly (ran) chosen ones. However, a rich zoo of phases emerges as equilibrium is approached: Asymmetric interactions (in the general sense of one species benefiting at the expense of another, see Supplementary Table 1) yield zonation, competitive exclusion, and other discontinuities in out-of-equilibrium situations, see Supplementary Fig. 4 and Supplementary Fig. 5e. Note that we could have found zonation with the alternative interactions of amensalism and repulsion since, for example, symmetric repulsive interactions in quantum gases do show spatial separations akin to zonation, see Supplementary Ref. [3].

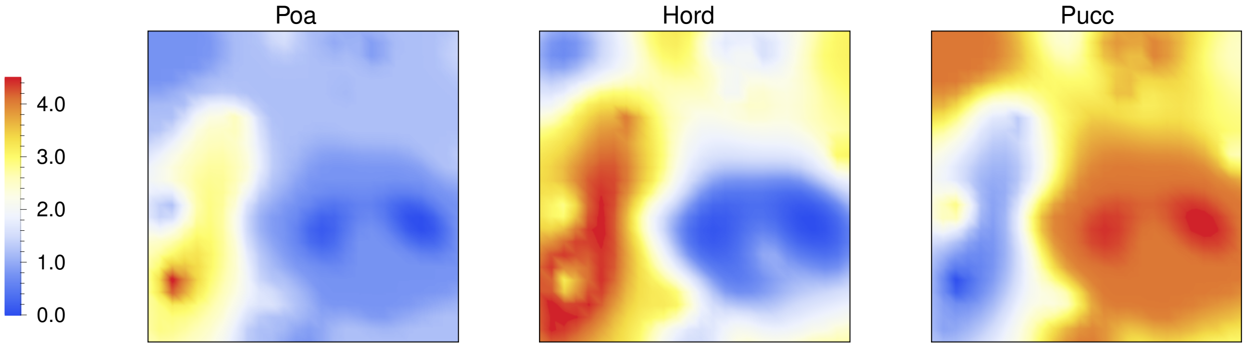

Supplementary Fig. 7. **Grass densities from an envelope model.** The absolute densities of Poa, Hord, and Pucc obtained for the salinity landscape of Fig. 4a in the envelope model that we employed in Fig. 4e and that is equivalent to the null model of the hypothesis that 'heterogeneous salinity has no effect on grass distributions'.

Notes on Fig. 5. In Refs. [36, 37], Veilleux reports the abundances of *Paramecium aurelia* (P), which feeds exclusively on Cerophyl, and *Didinium nasutum* (D), which feeds exclusively on P, under various conditions over periods of weeks. Supplementary Fig. 9 illustrates the three time series (out of five datasets in Ref. [36]) that exhibit the most unambiguous cyclic trajectories.

Arguably, only Supplementary Fig. 9a shows a more or less stable orbit over several periods and can thus be taken seriously as a predator–prey cycle. We deem the other two time series in Supplementary Fig. 9b/c not to qualify of having reached steady-state dynamics due to (i) the considerable disparity between the last and second-to-last complete cycle of the dataset shown Supplementary Fig. 9b, and (ii) the upward trend of P in Supplementary Fig. 9c.

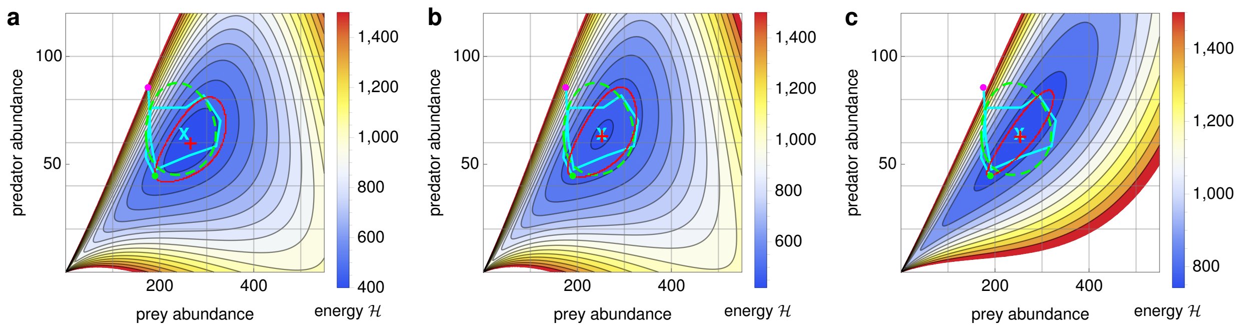

Supplementary Fig. 8. **A suitable fitting procedure for Veilleux's most stable predator–prey cycle.** We illustrate the DFTe hypersurface $\mathcal{H}(N_\text{P}, N_\text{D}) = E(N_\text{P}, N_\text{D})$ for equation (30) applied to the experimental setup of Supplementary Fig. 9a (the last fully visible cycle of the most stable predator–prey time series reported in Refs. [36, 37]), with parameters extracted from [37], see Supplementary Table 3. For a given DFTe equilibrium $E(\hat{\mathbf{N}})$ (red cross) we draw an equipotential line $E(\hat{\mathbf{N}}) + \mathit{\Delta}E$ (red line) on $\mathcal{H}$, with $\mathit{\Delta}E$ such that the amplitudes of the actual data cycle (cyan line) around the average abundances $\mathbf{N}^*$ (cyan cross) roughly match. The equipotential lines drawn here and in Supplementary Fig. 10 are meant to test the predictive power of the DFTe hypersurface away from its minimum. The magenta dot marks the first minimum of P's measured cycle, and the green dot marks the second minimum of D's cycle. **a**, $\mathcal{H}$ provides a reasonably accurate platform for the dynamics, with γ as the sole parameter for fitting $\hat{\mathbf{N}}$ to $\mathbf{N}^*$. **b**, With two fit parameters (γ,ν_PD), we obtain an even more satisfactory match between model and experiment, cf. Fig. 5. **c**, As one consistency check, we replace the common-sense choice of parasitism with amensalism and obtain a qualitatively different hypersurface: Comparing with **b**, we also find the equilibrium close to $\mathbf{N}^*$, but the DFTe trajectory is squeezed to such an extent that parasitism has to be regarded superior for modelling the predator–prey interaction mechanism. Moreover, only the hypersurface for parasitism permits noncyclic trajectories, which terminate as N_D vanishes, for strong enough 'excitations' $\mathit{\Delta}E$, that is, the predator population can collapse at high enough Cerophyl concentration while the prey survives, which is in line with experimental observations [37].

We aim at predicting the final cycle of all three datasets shown in Supplementary Fig. 9 with DFTe, while bearing in mind that any 'steady-state' predictions for the data of Supplementary Figs. 9b/c have to be taken with a grain of salt. With both species in suspension, we face a uniform situation like for Tilman's experimental setup studied above. Cerophyl concentrations are given in units of 1.8 g/litre. The unit of energy is fixed by $\zeta = 1$, see equation (7), and length by $A = 1000$.

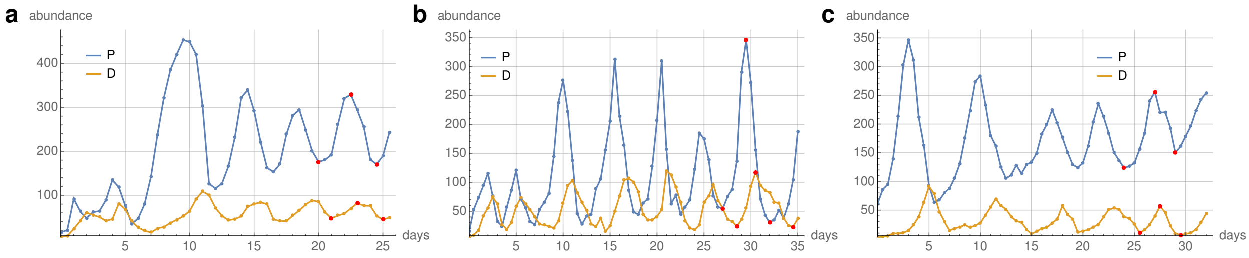

Supplementary Fig. 9. **Measured predator–prey cycles.** Veilleux's experimental data of the time evolution of abundances for *Didinium nasutum* (D) feeding on *Paramecium aurelia* (P) [36, 37]. Panels **a**, **b**, and **c** reproduce Figs. 14c, 11a, and 12a of Ref. [36]. The straight line segments guide the eye. We assume the steady-state abundance oscillations to cycle around reference equilibrium abundances $\mathbf{N}^* = (N_{\rm P}^*, N_{\rm D}^*)$, which we estimate as arithmetic means of the minima and maxima (red dots) of the last fully visible cycle of each time series, see Supplementary Table 3. We refrain from over-interpreting $\mathbf{N}^*$, which merely anchor our comparison between the trajectory on $\mathcal{H}$ and the experimental data. The last full cycle in panel **a** presents the most convincing steady-state trajectory among all three time series and serves as our primary benchmark.

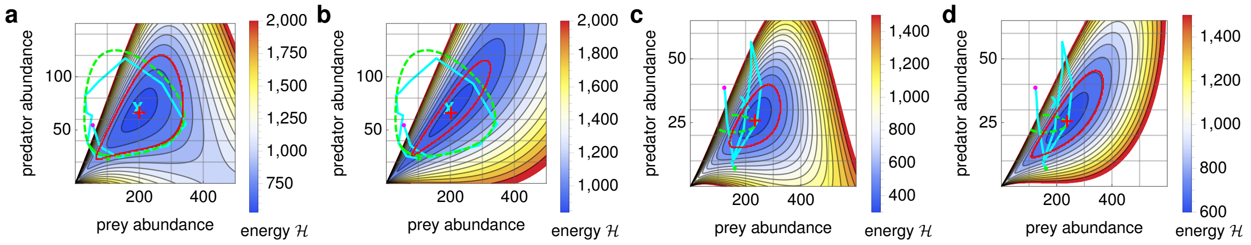

Supplementary Fig. 10. **DFTe predictions for two less stable predator–prey cycles.** Although the alleged steady-state cycles depicted in Supplementary Fig. 9b/c are not stabilised towards the end of the time series, we find a reasonable match with an equipotential line of the DFTe hypersurface $\mathcal{H}$ at least for panel **a**, which benchmarks DFTe against the data of Supplementary Fig. 9b with the parameters given in Supplementary Table 3. Panel **b** demonstrates that the same data cycle is less well captured if we choose amensalism instead of parasitism. The analogous calculations for Supplementary Fig. 9c are illustrated in panels **c** and **d**. Comparing panels **a** and **c**, we observe that a disturbance of the cycle can lead to the extinction of D more easily at higher Cerophyl concentration ρ_c. This is in line with the experiments reported in [37], where no stable cycles are found for $\rho_c > 0.75$. In view of the upward trend of P in Supplementary Fig. 9c near the end of the time series, we may argue that the equilibrated $N_{\rm P}^*$ is somewhat higher than what the cyan cross indicates, which would bring the DFTe equilibrium abundances in better agreement with the data. The Lotka–Volterra model (dashed green lines), see Supplementary Equations (28) and (29), performs well in panels **a/b** and fails in panels **c/d**. However, we do not want to over-interpret these observations, bearing in mind the experimental uncertainties pertinent to the steady-state character of the two setups of Supplementary Fig. 9b/c.

	panel in Supplementary Fig. 9 [Supplementary Fig. 8] {Supplementary Fig. 10}	interaction for DFTe	ρ_c	range of ν_{PD} (experiment)	manual removal of D	(γ, ν_{PD})	(N_P^*, N_D^*)	DFTe equilibrium abundances $(\hat{N}_P, \hat{N}_D)$ [relative deviation]
**a**	[**a**]	parasitism	0.5	3.0 ± 0.8	25%	(**29.2**,3.0)	(251,64)	(266,60) [6%]
	[**b**]	parasitism				(**30.75**,**2.7**)		(253,64) [0.5%]
	[**c**]	amensalism				(**1.97**,**3.0**)		(254,63) [1%]
**b**	{**a**}	parasitism	0.5	3.0 ± 0.8	0	(**56.5**,3.0)	(194,70)	(212,64) [9%]
						(**58.5**,**2.7**)		(201,68) [3.5%]
	{**b**}	amensalism				(**2.38**,**3.0**)		(203,68) [4%]
**c**	{**c**}	parasitism	0.375	9.1 ± 1.0	0	(**100**,9.1)	(196,33)	(246,25) [25%]
						(**108**,**8.1**)		(234,26) [19%]
	{**d**}	amensalism				(**1.95**,**9.1**)		(238,26) [21%]

Supplementary Table 3. **Fitted parameters and further details on the DFTe model.** The minima of the DFTe hypersurface, built from equation (30) with the best fits for the two parameters γ and ν_{PD}, are most consistent with Veilleux's data for a parasitic relation between predator and prey. As figure of merit we choose the relative deviation between the DFTe equilibrium abundances and (N_P^*, N_D^*), see Supplementary Fig. 9. Fitted parameters are typeset in bold. In the experiment represented by Supplementary Fig. 9a, D is manually removed from the suspension on a regular basis, corresponding to a $\Delta = 25\%$ reduction of D, which is implemented in equation (30) by the replacement $N_D \to N_D/(1-\Delta)$. If we refrain from optimising D's resource requirement ν_{PD} within its experimental uncertainty and stick with its central value, we still find the according one-parameter predictions of DFTe to match the data-based estimates (N_P^*, N_D^*) reasonably well. We tried (unsuccessfully) to improve the results for amensalism [encoded by the replacement $\frac{\gamma}{A^2} N_D N_P^2 \to \gamma N_P$ in equation (30)] by adjusting ν_{PD} away from its experimental central values. The DFTe equilibrium abundances correlate best with the experimental data depicted in Supplementary Fig. 9a, which incidentally represents the most convincing of the predator–prey cycles reported in Refs. [36, 37]. While the equilibrium abundances presented here do not justify making a strong case for either parasitism or amensalism, we settle the issue in favour of parasitism by observing the cycles on the DFTe hypersurface away from its minimum, see Supplementary Fig. 8 and Supplementary Fig. 10.

The Cerophyl resource for P is maintained at constant concentration ρ_c, while P itself serves as resource for D. The data in Ref. [37] show that the abundance of P in monoculture scales linearly with ρ_c ($N_{\mathrm{P}} = 500$ for $\rho_c = 0.5$), such that the Cerophyl requirement of P is $\nu_{cP} = 1$. The prey requirement of D also depends on ρ_c with more (starved) prey individuals required at lower Cerophyl concentration. All DFTe input parameters are extracted from the data reported in Refs. [36, 37] and listed in Supplementary Table 3. The predator–prey relation between the two species is certainly beneficial for D and harmful for P, such that parasitism is the natural choice for the interaction functional, see Supplementary Table 1. That is, excluding self-interaction and installing D as the logical beneficiary of the interaction, we set up the competition matrix $\gamma_{ss'} = \gamma\,\delta_{sD}\,\delta_{s'P}$ with only one nonzero component. Disregarding the experimental uncertainties in the resource requirements of P and D, we are left with the interaction strength γ as sole fit parameter in equation (30). In Supplementary Table 3 we report $\gamma = 29.2$ for Supplementary Fig. 9a to yield the best match between $\hat{\mathbf{N}}$ and the reference equilibrium abundances $\mathbf{N}^*$. The according DFTe hypersurface in Supplementary Fig. 8a not only produces a reasonable match between the abundances predicted by DFTe and $\mathbf{N}^*$, but also between the last cycle of Supplementary Fig. 9a and an equipotential line (red) of appropriate amplitude on $\mathcal{H}$. Optimising the resource requirement of D within its experimental range of uncertainty, we obtain the hypersurface in Fig. 5 and Supplementary Fig. 8b, which shows an even better match between data and DFTe calculation. The comparison with the squeezed equipotential line in Supplementary Fig. 8c suggests that, as common sense demands, parasitism outperforms amensalism in describing the predator–prey oscillations with DFTe.

The equipotential lines drawn in Supplementary Fig. 8 and Supplementary Fig. 10 are meant to test the predictive power of the DFTe hypersurface away from its minimum. A quantitative time-dependent version of DFTe, in particular the functional form of the resource energy (which is simply quadratic in equation 9), has to be informed by dynamic variables and characteristic time scales such as birth- and death rates, time delays in interaction mechanisms (for example, due to metabolism or foraging), environmental variability and so forth. The predator–prey cycle can approximately be regarded as an ellipse, which is determined by four parameters (position, orientation, and eccentricity). Our use of three fit parameters (ν_{PD},γ, and ΔE) would thus not suggest a particularly powerful approach. However, the underlying functional in equation (30) provides causal understanding of the intra- and inter-specific relations and follows from the universal DFTe functional in equation (9), which we proved to be applicable to a wide range of systems.

Furthermore, the three-parameter DFTe equipotential lines in Fig. 5, Supplementary Fig. 8, and Supplementary Fig. 10 are of a quality similar to the Lotka–Volterra predictions from

$$\frac{\mathrm{d}N_\mathrm{P}}{\mathrm{d}t} = r_\mathrm{P}\,N_\mathrm{P} - a_\mathrm{PD}\,N_\mathrm{P}\,N_\mathrm{D} \tag{28}$$

$$\frac{\mathrm{d}N_\mathrm{D}}{\mathrm{d}t} = b_\mathrm{PD}\,N_\mathrm{P}\,N_\mathrm{D} - m_\mathrm{D}\,N_\mathrm{D}, \tag{29}$$

where six parameters yield best fits to the experimental cycles of $(N_\mathrm{P}, N_\mathrm{D})$ in Supplementary Fig. 9 for

Supplementary Fig.	$N_\mathrm{P}(0)$	$N_\mathrm{D}(0)$	a_PD	b_PD	m_D	r_P
9a	171.119	61.5892	0.01962	0.005960	1.41462	1.25682
9b	46.1652	34.5678	0.02552	0.005417	0.67998	1.59854
9c	108.445	22.0646	0.09895	0.001345	0.20560	2.41680

Here, $N_\mathrm{P}(0)$ and $N_\mathrm{D}(0)$ are the fitted initial prey and predator abundances, respectively, t is time, r_P is the intrinsic growth rate of the prey, a_PD is the per-predator per-prey predation rate, b_PD is equal to a_PD times a conversion factor, and m_D is the mortality rate of predators.

Notes on Fig. 6. In the following, we provide a detailed account of the DFTe input and the resulting density distributions shown in Supplementary Fig. 13 below. The according simulations, which eventually lead to Fig. 6, always include dispersal ($E_\mathrm{dis}[\mathbf{n}]$), whose trade-off against resources ($E_\mathrm{Res}[\mathbf{n}]$) creates an intuitively accessible base line for studying more complex (sub-)systems that include environments ($E_\mathrm{env}[\mathbf{n}]$) and interactions ($E_\gamma[\mathbf{n}]$).

Comparing the Tree distribution n_5 in Supplementary Fig. 13a (‘**a**’ in the following) with the density in Supplementary Fig. 12e, we observe the effect of the inter-species competition over resources. Without any further interactions, all three species $\{1(\text{Fungus}), 5(\text{Tree}), 6(\text{Grass})\}$ have the same ability to exploit the resources (since $\tau_s = 0.01$, see Supplementary Table 4), resulting in $n_5 = n_6$ (both species have the same resource requirements). The Fungus does not require sunlight (ρ_5) and finds its primary niche near the western boundary, where (i) water and nutrient occur at high density and (ii) the Tree and Grass exert little competition pressure due to their small densities induced by a lack of sunlight. Although the Tree and Grass are identically parameterised in **a** (prior to the inclusion of environments and interactions), they are still distinguishable species because the (**n**-)nonlinearity of equation (31) implies different energies for (i) two species with

independent variables n_5 and n_6 versus (ii) one 'effective species' comprised of the sum $n_5 + n_6$. All three species have the same density profiles in the south-eastern quadrant, where water is their limiting resource. Both Tree and Grass are limited by sunlight in the west and thus must let the Fungus take advantage of the underutilised resources of water and nutrient, cf. ρ_{lim} in Supplementary Fig. 12. For the central ring region we observe that the Tree and Grass equally divide their limiting resource (sunlight, at densities of $\sim 9 \ldots 15$) and therefore aim at extracting an equivalent amount of nutrient. This puts competition pressure on the nutrient-limited Fungus, resulting in $n_1 \approx 4 < n_5 = n_6 \approx 6$, despite Tree and Grass being constrained by three resources while the Fungus only requires two.

$\left(\Gamma^{(a)}_{ss'}\right)$	$\left(\Gamma^{(r)}_{ss'} + \Gamma^{(m)}_{ss'}\right)$	$\left(\Gamma^{(c)}_{ss'} + \Gamma^{(p)}_{ss'}\right)$	(ν_{ks})
$\begin{pmatrix} 0&0&0&0&0&0&0 \\ 0&0&0&0&0&0&0 \\ 0&0&0&0&0&0&0 \\ 0&0&0&0&0&0&0 \\ 0&0&-1&0&0&1&0 \\ 0&0&0&-1&0&0&0 \\ 0&0&0&0&0&0&0 \end{pmatrix}$	$\begin{pmatrix} 0&0&0&0&0&1&0 \\ 0&0&0&0&0&0&0 \\ 0&0&0&0&0&0&0 \\ 0&0&0&0&0&0&0 \\ -1&-1&0&0&0&0&0 \\ 0&0&0&0&0&0&0 \\ 0&0&0&0&0&0&0 \end{pmatrix}$	$\begin{pmatrix} 0&0&0&0&0&0&0 \\ 0&0&0&0&0&0&0 \\ 1/2&0&0&0&0&0&0 \\ 1/2&0&0&0&0&0&0 \\ 0&0&0&0&0&0&0 \\ 0&0&0&0&0&0&0 \\ 0&1/2&1/2&0&0&0&0 \end{pmatrix}$	$\begin{pmatrix} 0&0&1&0&0&0&0 \\ 0&0&0&0&0&0&5 \\ 0&0&0&0&0&0&1 \\ 1&1&1&1&1&1&0 \\ 0&0&0&0&1&1&0 \\ 1&1&1&1&1&1&0 \end{pmatrix}$

Supplementary Table 4. **Synthetic community: Interactions, resource consumption, and dispersal.** Each of the inter-species bipartite interactions, indicated in Fig. 6 with arrow heads that identify the primary direction of influence, is introduced with contact-type interaction kernel $\gamma^{()}_{ss'}(\mathbf{r}, \mathbf{r}') = 0.01\, \delta(\mathbf{r} - \mathbf{r}')\, \Gamma^{()}_{ss'}$. The matrices $\left(\Gamma^{()}_{ss'}\right)$ encode commensalism/amensalism (a), repulsion (r), mutualism (m), asymmetric competition (c), and parasitic interaction (p) that accompany predator–prey relations, see Supplementary Table 1. For example, $\left(\Gamma^{(c)}_{ss'} + \Gamma^{(p)}_{ss'}\right)$ favours $s = 4$ over $s' = 1$ and lets $s = 3$ benefit at the expense of its prey $s' = 1$. To model a subset of species and resources, we simply extract the according submatrices of the matrices shown here. The first three rows of the resource requirements (ν_{ks}) represent the prey species $s = 1$ and $s = \{2, 3\}$, consumed by the predators $s = 3$ and $s = 7$, respectively. In declaring the prey densities of Deer ($\rho_2 =]n_2[$) and Pig ($\rho_3 =]n_3[$) as separate resources for the Cat, we stipulate its reliance on each, cf. equation (7). If either prey could maintain the Cat, the combined resource density $n_2 + \omega\, n_3$ were to replace $\rho_2 = n_2$ and $\rho_3 = n_3$, with a ratio ω to be specified. The rows 4–6 of (ν_{ks}) identify the species' requirements of the fixed equilibrium resource distributions $\rho_{4\text{–}6}$ depicted in Supplementary Fig. 11. We ensure that resources can be exploited to a significant degree by choosing moderate (with respect to the energy scale set by $\zeta = 1$ in equation (7)) dispersal pressures $\boldsymbol{\tau} = 0.01 \times (1, 2, 2, 0.3, 1, 1, 0.1)$ that penalise large local population densities only slightly.

As the environments are added in **b**, the Fungus distribution (and, consequently, its abundance N_1) changes only marginally since its density in the environmentally hostile regions is low anyway. In contrast, V^{env}_5 expels the Tree from the hostile central region, allowing the Grass to invade.

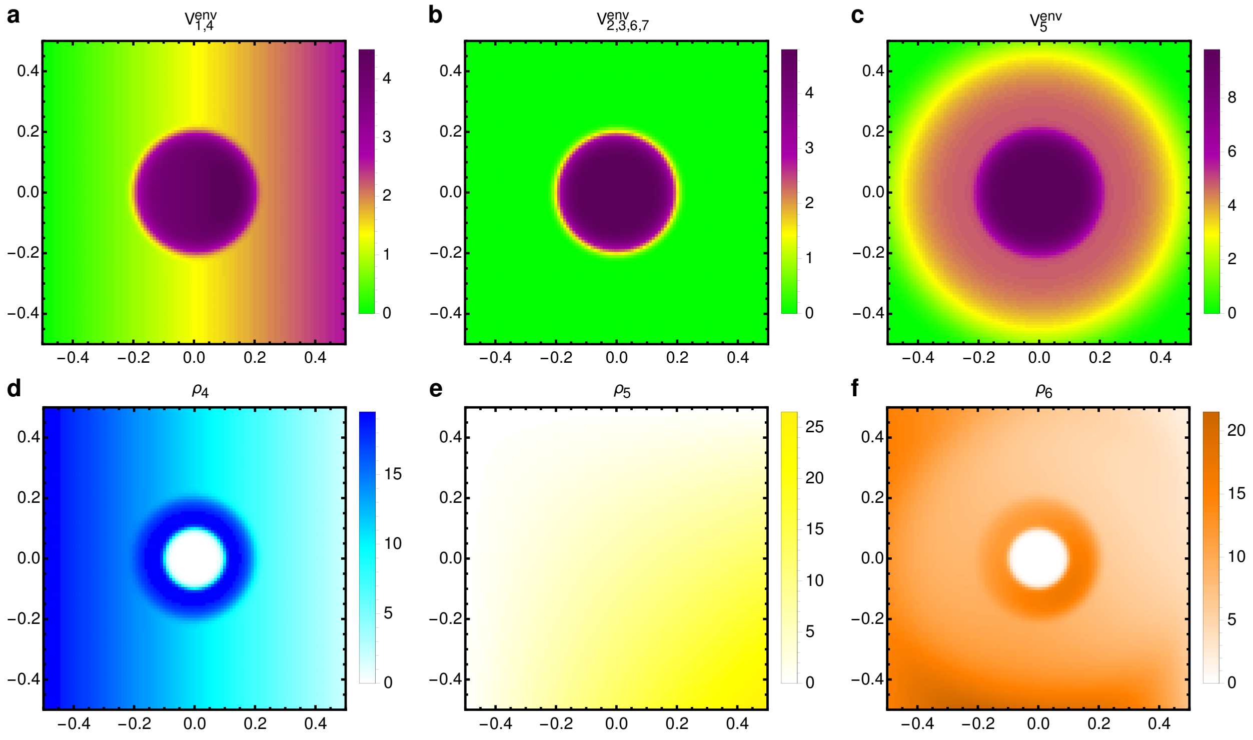

Supplementary Fig. 11. **Synthetic community: Environments and resources.** **a**–**c**, The species-specific environments $V^{\text{env}}_{1,4}$, $V^{\text{env}}_{2,3,6,7}$, and V^{env}_{5} (all scaled with a factor of 100), from favourable (green) to hostile (black). **d**–**f**, The resource distributions ρ_4 ('water') and ρ_5 ('sunlight') are correlated with ρ_6 ('generic nutrient'). Choosing $\sigma = -4$ in equation (31), we assign the nonlimiting resources a minor but nonzero role relative to the limiting resources, see equation (8). We declare that the Trees' environmental strain V^{env}_{5} represents the deforestation stress, which is most detrimental in the central region. The central disk-shaped region is void of accessible resources $\rho_{4,6}$, for example, due to human presence.

This includes the central ring region, where the Grass environment V^{env}_{6} is also hostile. There, the Grass density even exceeds that of the neutral (uniformly zero) environment employed in **a**. This phenomenon of an increased density in a less favourable habitat originates in the trade-off between all DFTe energy components of all species and may be perceived as counterintuitive prior to our quantitative assessment.

The picture of a Grass-dominated landscape changes drastically as we complete the subsystem of the species $\{1, 5, 6\}$ by introducing their bipartite interactions in **c**. The combined resource-environment-dispersal constraints on densities are modified by the mutualism between Tree and Fungus, which permits higher densities of both species and tends to align them. As a result, Grass comes under considerable pressure due to (i) the amensalism where Trees exist and (ii) the repelling Fungus. While the Fungus-Grass repulsion affects both species in a symmetric way (and allows Grass to expel the Fungus from its low-density regions), the Tree-Fungus alliance renders Grass competitively inferior to the Fungus in regions of high Tree density.

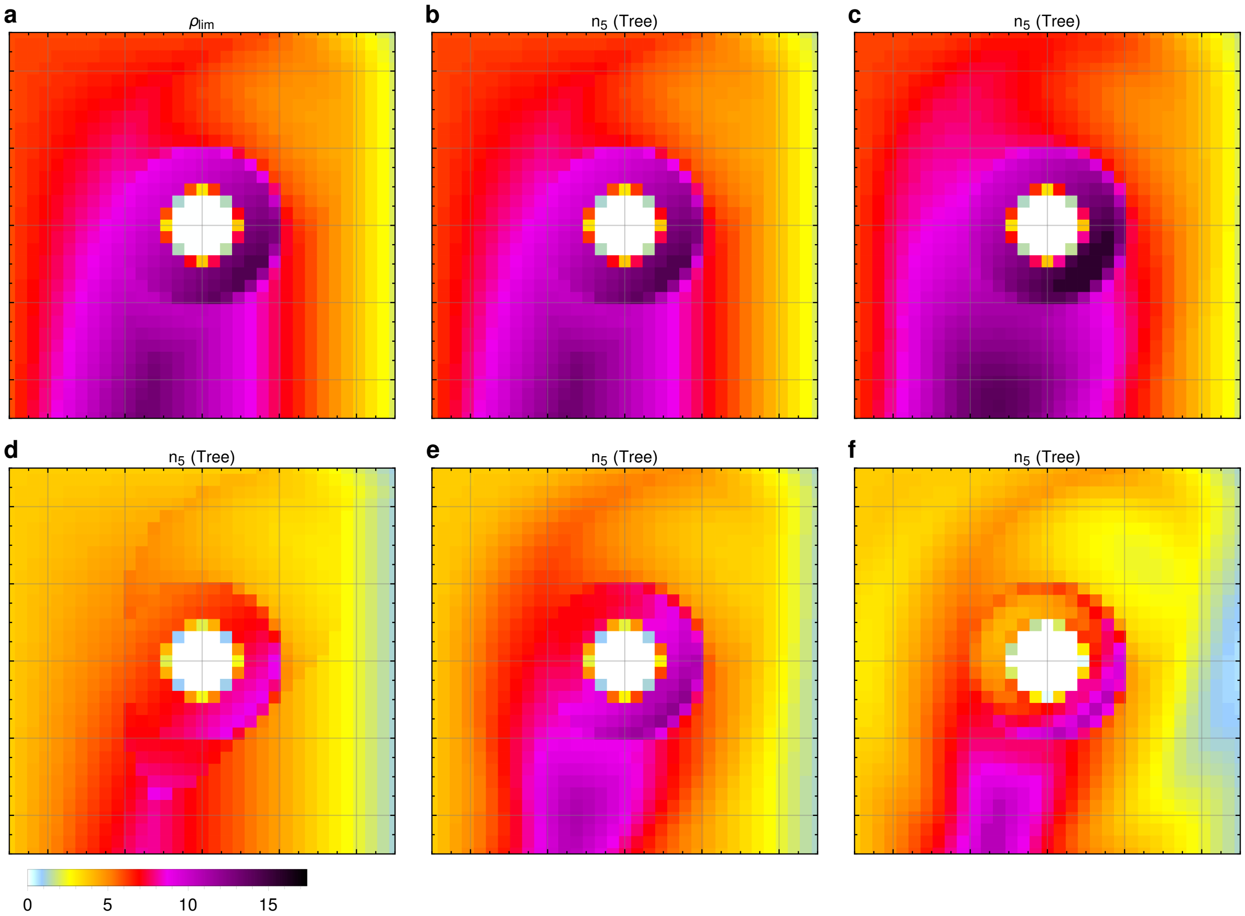

Supplementary Fig. 12. **Synthetic community: Parameterisation of the Tree.** We illuminate the interplay between dispersal, environment, and resources by modelling a single species (Tree, $s = 5$) with exclusive access to all resources. The color code applies to all panels. **a**, The locally limiting resource density $\rho_{\mathrm{lim}}(\mathbf{r}) = \min_{k=4,5,6} \rho_k(\mathbf{r})$ for the Tree, which requires equal amounts of $\rho_{4,5,6}$. **b**, The equilibrium density distribution n_5, with $\sigma = -1000$, $\tau_5 = 10^{-8}$, and uniform environment (V_5^{env} is replaced with zero), closely aligns with ρ_{lim} since (i) the dispersal pressure is essentially zero and (ii) the nonlimiting resources have negligible effect. **c**, The visible disparity to **b** is due to the smearing effect of the nonlimiting resources, triggered by setting $\sigma = -4$, which relaxes the constraints imposed by the limiting resources and permits higher overall consumption. **d**, Increasing τ_5 to 0.01 while keeping $\sigma = -1000$, we reveal the adverse effect of the dispersal pressure from local conspecifics. **e**, The density for our generic choices $\tau_5 = 0.01$ and $\sigma = -4$, where the dispersal pressure is counteracted by the density-increasing effect of the nonlimiting resources. **f**, The introduced environment V_5^{env} (see Supplementary Fig. 11) expels the Tree from hostile regions—to an extent that is determined by balancing (through the minimisation of the DFTe energy) the according environmental pressure against (i) the dispersal pressure and (ii) the conspecific resource competition.

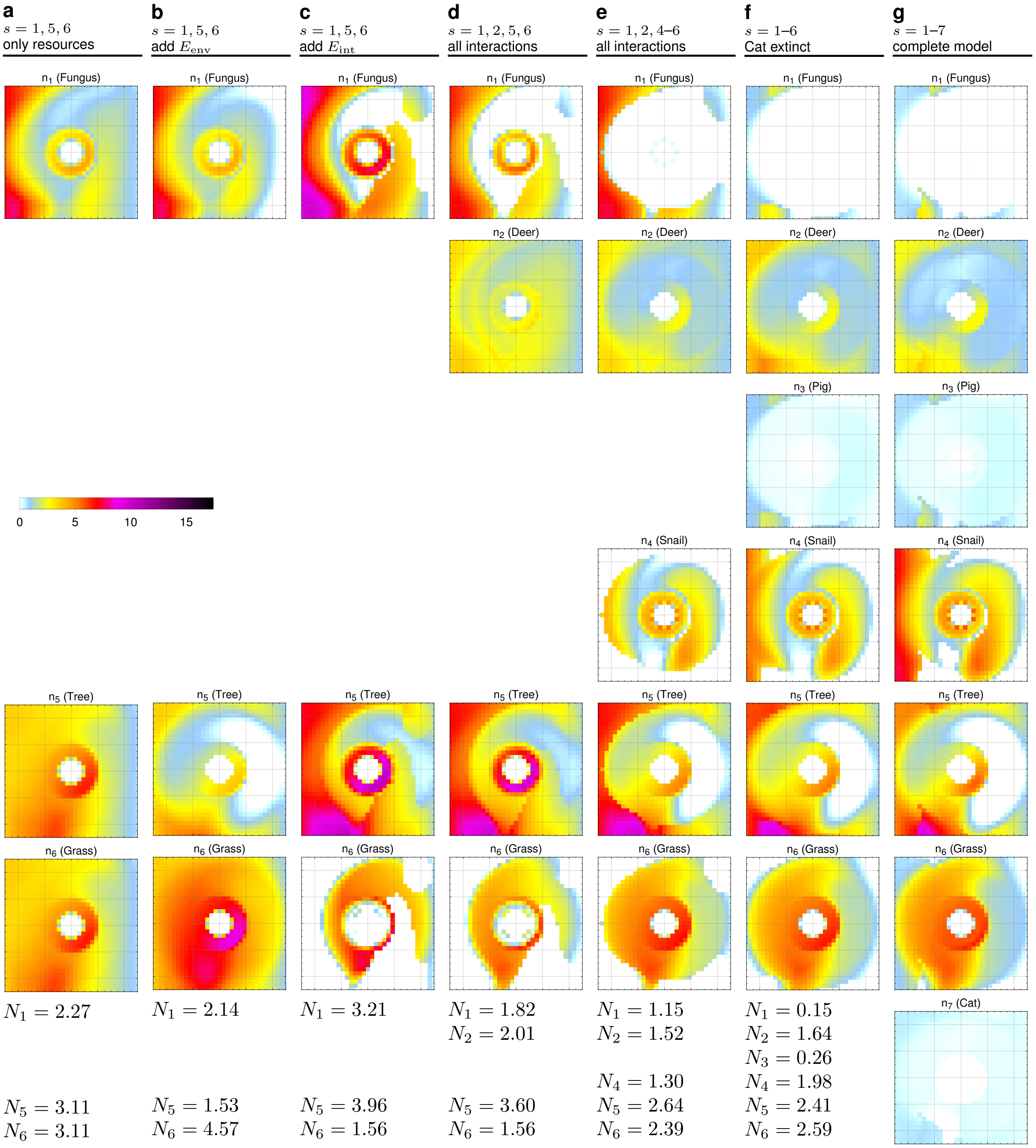

Supplementary Fig. 13. **Building the synthetic community bottom-up.** Each column (**a**–**g**; shared colour code) specifies the involved species and the energy components that are successively added to E_{dis}; see Supplementary Notes for a detailed interpretation of **a**–**g**. The transition from **g**, with abundances $\mathbf{N} = \{0.13, 1.26, 0.29, 2.53, 2.04, 2.69, 0.19\}$, to **f** amounts to an artificial extermination of the apex predator (Cat), that is, the absence of the Cat in **f** is not the result of competitive exclusion but of external intervention. The according relative redistributions of $s = 1$–6 are spatially resolved in Fig. 6b. The main insights are (i) the decline of Grass and Snail populations, (ii) increased abundances of their heterospecifics through feedback loops, (iii) knowledge on how the inter-species mechanisms, coupled with the environmental constraints, lead to these results, and (iv) which specific actions to take for expanding or helping reintroduce the Cat population (see Supplementary Fig. 14).

Grass can coexist with both the Tree and the Fungus (where their densities are low) and even dominates in regions that are both poor in resources and environmentally hostile for the Tree. However, we predict competitive exclusion of Grass from Fungus territory, except where (prior to interactions) the Fungus density is very low and the Grass density is very high. This balance between species, enforced at the DFTe energy minimum, also gives rise to sharp interfaces between Grass and the other two species, contrasting with the smooth (noninteracting) densities in **a** and **b**. Note that this zonation pattern is equilibrated, unlike the transient zonation states in Supplementary Fig. 4. The zonation originates in the Fungus-Grass repulsion but is transferred to the Tree distribution via the Tree-Fungus mutualism. More of such discontinuities emerge and transform as we keep adding species and their interactions.

In **d** we introduce the Deer, who owes its relatively uniform distribution to the lack of adversarial interactions like those between Grass and Fungus. Although all heterospecifics suffer from the additional competitor over resources, the Tree nearly makes up for the loss through the added mutualistic interaction. That is, the Deer's resource consumption primarily lowers the densities of the Fungus and (to a lesser extent) of Grass, which alleviates both species' conspecific dispersal pressure and the Fungus-induced repulsive pressure on Grass. The result is a less pronounced zonation between Grass and its competitors. Although the Grass density decreases, its land cover increases where the Fungus recedes. In summary, the global Tree and Grass abundances are roughly maintained, and the main impact of introducing the Deer is the (a priori surprising) decline of the trophically distant Fungus.

The Snail, introduced in **e**, has the same habitat preference and resource requirements as the Fungus. As expected, the resulting intense competition between the two species is decided in favour of the superior Snail, see Supplementary Table 4, which expels the Fungus from favourable regions. However, the mutualistic trio with the Tree and Deer protects the Fungus against the Snail in areas of high Tree density. While the competition via asymmetric interaction also incurs a cost for the Snail, albeit lower than for the Fungus, the Snail more than compensates this disadvantage (compared to the mutualistically bolstered Tree and Deer) through its low dispersal pressure, which enables a more efficient exploitation of resources. This benefits the Snail particularly in the heavily contested central ring region, where a high Grass density (implied by the excluded Fungus and diminished Tree) supports the Snail commensalistically. Both Deer and Tree, mutualistically

connected to the Fungus, suffer from lowered Fungus density. Aside from occupying former Fungus territory, the Snail can therefore carve out the two ‘circle segments’ beside the central ring region, where all the other species are relatively low in density (before and even more so after the Snail’s introduction) and thus offer little resistance. Although Grass does not benefit from the Snail through a direct interaction, the implications of the Snail’s introduction ripple through the community and make Grass the main beneficiary, particularly where the Fungus and the Tree recede along with their competitive pressure.

The Pig, introduced as predator in **f**, relies on and thus aligns with the Fungus. The cost (E_{Res}) of nonzero Pig density in regions void of Fungus can only be absorbed to a minor degree by the other energy components (in particular the nonlimiting resources), such that the Pig is largely restricted to Fungus habitat. The predation has several implications across the community. As expected, the Fungus density is severely diminished globally, and more of the resource pools ρ_4 and ρ_6 in the affected regions become available to the rest of the community. The Pig consumes only a small part of this excess $\mathit{\Delta}\rho_{4,6}$, and both the Snail and the Grass do not benefit from the released resources since their habitat hardly overlaps with that of the Fungus. The Tree loses most of its mutualistic Fungus support, most visibly in regions of high Tree density, which outweighs the positive impact of $\mathit{\Delta}\rho_{4,6}$. The alleviated amensalistic pressure from the Tree not only allows Grass to invade into former Tree territory (aided by the diminished repulsion from the Fungus), but also releases additional resources, incidentally in some of the preferred habitat of the Deer and Snail. The combination of these direct and indirect mechanisms thus leaves the Deer, the Grass, and especially the Snail as the beneficiaries of the Pig’s introduction. While the positive impact of the Pig on Grass and Snail are in line with an intuitive examination of the interactions illustrated in Fig. 6, the growth in the Deer population, mutualistically connected to the declining Tree, may come as a surprise if not all the ecosystem ingredients are taken into account quantitatively.

In **g**, we finally introduce the Cat as the apex predator, with an equilibrated density distribution that is maintained by five Deer and one Pig per individual, see Supplementary Table 4. The Cat closely aligns with the Pig, except where the Deer is the limiting prey that alleviates the predation pressure on the Pig (such as the eastern circle segment, where the ratio between Deer and Pig falls below 5:1) by making the Pig a nonlimiting resource that is affected only marginally due to $\sigma = -4$ for the resource weights. The Fungus distribution correlates with the Pig population not because of a causal relationship—if anything, we would expect the Fungus to benefit as the Pig becomes

prey for the Cat. The Pig distribution follows the Fungus distribution as per their predator–prey relationship, and the Fungus is in decline because (i) the Tree is receding and (ii) the Snail establishes a stronger foothold globally, also in the western Fungus habitat. The source of this cascade of changes in the community is the heavy predation of the Deer, accompanied by a decline of the mutualistically connected Tree, which therefore decreases its amensalistic pressure on Grass and its support for the Fungus. The Snail then benefits from an elevated Grass density as well as the released resources and puts additional competition pressure on the Fungus, such that the Tree loses even more support. This negative (positive) feedback loop between Tree, Deer, and Fungus (Grass and Snail) finally stabilises at the DFTe energy minimum with the density distributions in **g**. By exterminating the Cat (that is, transiting from **g** to **f**), we affect the community the opposite way—the time-independent DFTe energy functional in equation (31) does not include history and therefore no hysteresis. In Fig. 6 we depict the relative changes $\Delta n_s(\mathbf{r}) = \left(n_s^{\mathbf{f}}(\mathbf{r}) - n_s^{\mathbf{g}}(\mathbf{r})\right) \Big/ \max\left\{n_s^{\mathbf{g}}\right\}$ of the equilibrium density distributions for the species $s \in 1$–6.

If we were to strengthen the Cat population, we could, for example, reduce deforestation globally. This benefits (i) the Deer and (ii) the Pig (through enhancing the primary Fungus habitat, see first panel of Supplementary Fig. 14a). The quantitative impact of

$$V_5^{\text{env}} \to \tilde{V}_5^{\text{env}} = \frac{1}{2} V_5^{\text{env}} \tag{30}$$

is shown in Supplementary Fig. 14b/c, and the relative density differences to column **g** of Supplementary Fig. 13 are shown in Fig. 6c.

Approximating realistic long-range interactions by contact-type (i.e., zero-range) interactions, as we implicitly do in our synthetic food web example, is only justified if the simulation area A is sufficiently coarse-grained into areas $A' < A$, such that regions beyond the focal area A' (which contains the focal position $\mathbf{r}'$) have negligible interaction effect on the density $n(\mathbf{r}')$:

$$\mathcal{I}_A = \int_A \mathrm{d}\mathbf{r}\, \gamma(\mathbf{r}', \mathbf{r})\, n(\mathbf{r}) \approx \int_{A'} \mathrm{d}\mathbf{r}\, \gamma(\mathbf{r}', \mathbf{r})\, n(\mathbf{r}). \tag{31}$$

This requirement crucially depends on the interaction kernel γ, whose characteristic length scales relate to A. As an illustration, we take a Yukawa potential $\gamma(\mathbf{r}', \mathbf{r}) = \gamma \exp[-|\mathbf{r} - \mathbf{r}'|/l]/|\mathbf{r} - \mathbf{r}'|$ with characteristic length scale $l = 0.01\mathcal{L}$, focal position $\mathbf{r}' = 0$, and constant densities $n(\mathbf{r}) = n$ in a disk-shaped area $A = 2\pi\mathcal{L}^2$, such that $\mathcal{I}_{A'=2\pi R'^2} = 2\pi\gamma n l\,(1 - \exp[-R'/l])$. Then, demanding $\mathcal{I}_{A'}/\mathcal{I}_A > 95\%$, i.e., obeying Supplementary Equation (31) accordingly, requires us to choose $R' > \mathcal{L}/34$. Note that A in Fig. 6 is subdivided into parcels of $(\mathcal{L}/33)^2$.

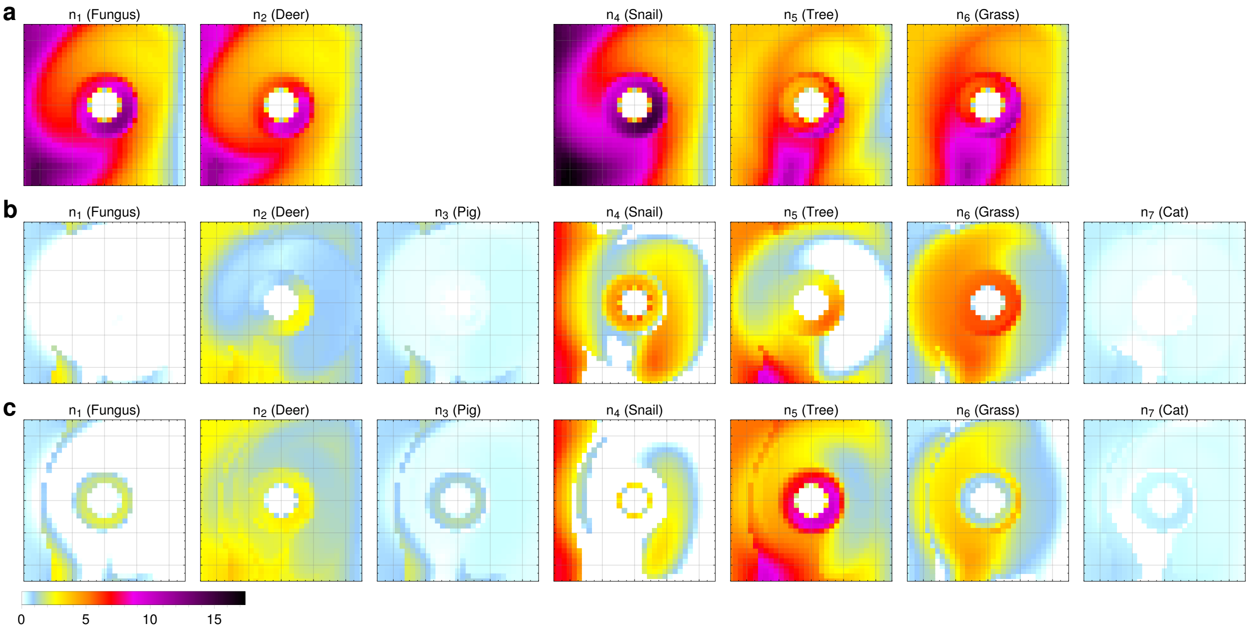

Supplementary Fig. 14. **Synthetic community: Species in isolation, at equilibrium, and in modified environments.** Interactions can considerably transform species distributions and decorrelate them from the resource distributions. The colour code applies to all panels. **a**, Distributions of the species $s \in \{1, 2, 4–6\}$ in isolation, that is, in absence of all $s' \neq s$ and solely constrained by the resource distributions and the s-specific environment. We disregard the predators Pig and Cat, which rely on prey resources and thus inherently require inter-species interactions. While Snail and Fungus perceive the same environment and require the same resources, the Snail is a more efficient consumer due to its lower dispersal pressure, which results in higher densities. The Deer experiences a favourable environment except in the central region, but its high dispersal pressure leads to lower densities overall. The Tree and Grass only differ in their environment, see Supplementary Table 4 and Supplementary Fig. 11. **b**, Distributions (identical to column **g** of Supplementary Fig. 13) for the complete interacting community, including predation. **c**, We expand the Cat population by cutting the deforestation stress for the Tree in half (see Supplementary Equation (30)). Evidently, this measure not only increases the Cat abundance from $N_7 = 0.19$ to $N_7 = 0.26$, but affects the entire community significantly. The relative difference between **b** and **c** is depicted in Fig. 6c.

Notes on Fig. 7. We obtained the reference density data shown in Fig. 7 and Supplementary Fig. 15 as follows. While we considered all data from the eight censuses carried out between 1982 and 2015, we omitted about 700 (out of more than 400000) entries of the raw data where consistency checks (same tree–ID & same tag & same coordinates) for individual trees failed. After calculating the average basal area $\langle B(t) \rangle$ of each individual tree t across the eight censuses, we assigned the abundance $N_s(q)$ of a species s in quadrat q (with area $20\,\mathrm{m} \times 20\,\mathrm{m}$) of the fifty–hectare plot by accumulating all $\langle B(t) \rangle$ that belong to a tree of species s located within q and so identify the $S = 20$ most abundant species in order of decreasing abundance from $s = 1$ (*Quararibea asterolepis*) to $s = 20$ (*Virola sebifera*), see Supplementary Table 5. We interpolated the measurements of the two

environmental variables of altitude and pH-level as well as the eleven resources (Al, B, Ca, Cu, Fe, K, Mg, Mn, P, Zn, N) to obtain their values at the centres of the quadrats q. By averaging over multiple quadrats q, we obtained the environmental base potentials $V^{\mathrm{alt}}(\mathbf{r})$ and $V^{\mathrm{pH}}(\mathbf{r})$ as well as the resource densities $\rho_k(\mathbf{r})$ for $k = 1, \ldots, 11$ with centre coordinates $\mathbf{r}$ of $9 \times 5 = 45$ larger quadrats Q of the $1000\,\mathrm{m} \times 500\,\mathrm{m}$ plot. This coarse-graining tempers the effects of stochastic processes on local densities and reduces computational cost. The bottom bar charts in Fig. 7a and in Supplementary Fig. 15 show the resulting reference densities $n_s^{\mathrm{ref}}(\mathbf{r})$ for $s = 1, \ldots, 20$.

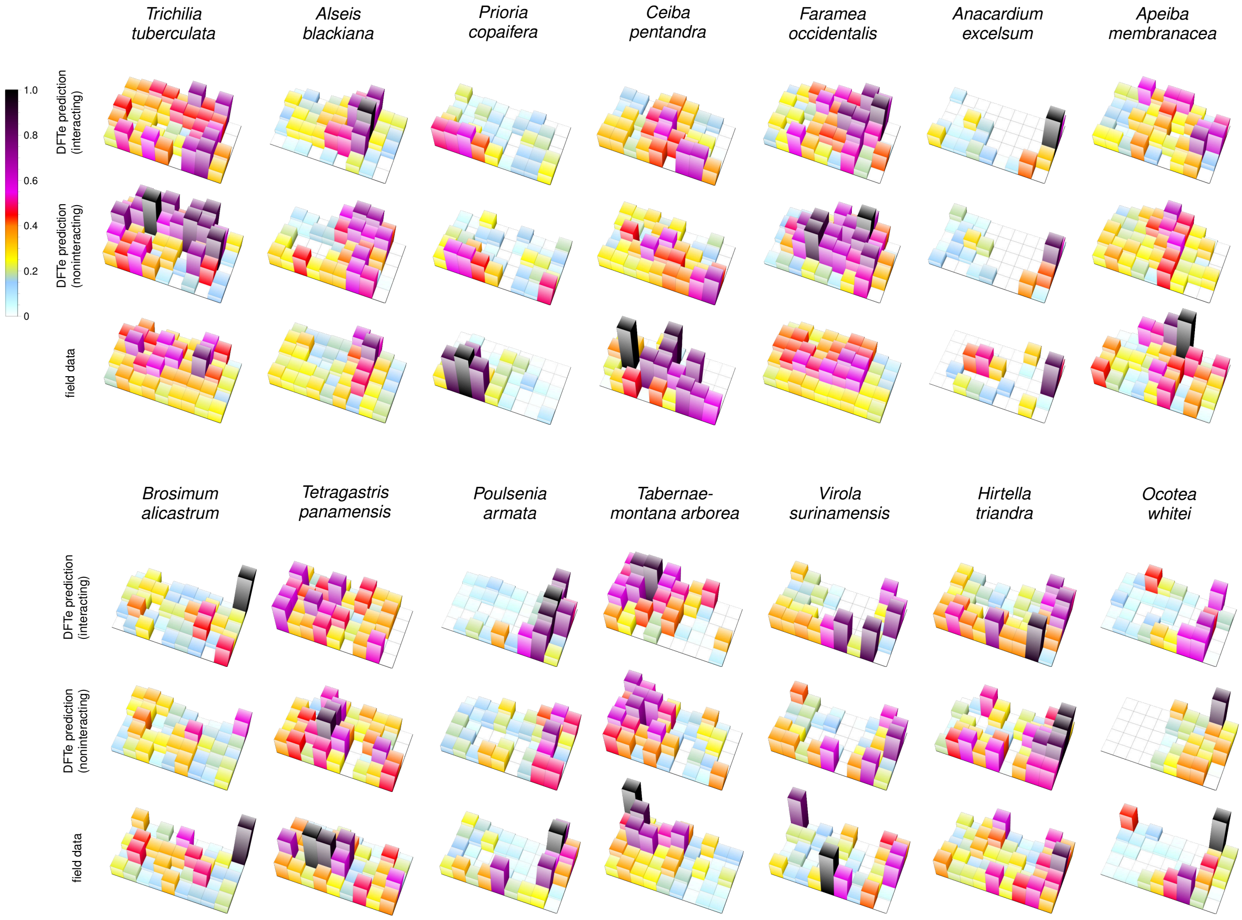

Supplementary Fig. 15. **DFTe fit results and BCI reference data.** As in Fig. 7, but for the remaining fourteen species $s = 2, 3, 5\text{–}7, 9\text{–}11, 13\text{–}15, 17\text{–}19$, see Supplementary Table 5: We obtained the DFTe densities $\tilde{n}_s$ ($\tilde{n}_s^0$) in the top (centre) charts, which represent the average fractional basal area over the eight censuses of basal area since 1982 [42], from fitting the full DFTe energy functional with (without) inter-species interactions to the reference densities n_s^{ref} in the bottom charts. The density values shown in the three bar charts for each species s are rescaled to the unit interval via division by the maximum of $\tilde{n}_s$, $\tilde{n}_s^0$, and n_s^{ref}.

species index s	species name	ForestGEO identifier	$\mathbf{N}^{\text{ref}}$ [m^2]	ξ	ξ^0
1	*Quararibea asterolepis*	QUARAS	108.3	0.791	0.789
2	*Trichilia tuberculata*	TRI2TU	95.7	0.792	0.790
3	*Alseis blackiana*	ALSEBL	69.5	0.791	0.793
4	*Hura crepitans*	HURACR	65.7	0.791	0.789
5	*Prioria copaifera*	PRI2CO	64.7	0.791	0.788
6	*Ceiba pentandra*	CEIBPE	59.8	0.791	0.789
7	*Faramea occidentalis*	FARAOC	57.4	0.795	0.791
8	*Jacaranda copaia*	JAC1CO	38.5	0.791	0.792
9	*Anacardium excelsum*	ANACEX	32.8	0.801	0.794
10	*Apeiba membranacea*	APEIME	32.6	0.792	0.789
11	*Brosimum alicastrum*	BROSAL	30.7	0.792	0.803
12	*Beilschmiedia pendula*	BEILPE	30.6	0.791	0.788
13	*Tetragastris panamensis*	TET2PA	30.3	0.793	0.807
14	*Poulsenia armata*	POULAR	29.1	0.793	0.791
15	*Tabernaemontana arborea*	TAB2AR	28.4	0.793	0.788
16	*Cavanillesia platanifolia*	CAVAPL	24.7	0.793	0.793
17	*Virola surinamensis*	VIROSU	24.6	0.791	0.789
18	*Hirtella triandra*	HIRTTR	22.5	0.794	0.790
19	*Ocotea whitei*	OCOTWH	22.2	0.793	0.788
20	*Virola sebifera*	VIROSE	21.0	0.794	0.797

Supplementary Table 5. **Tree species modelled in the case study of Fig. 7.** The twenty most abundant species in terms of the average total basal areas $\mathbf{N}^{\text{ref}}$, as extracted from the eight censuses of the 50-ha plot on Barro Colorado Island [42]. We maximised the overlap ξ (ξ^0) between abundance data and DFTe simulation with (without) bipartite interactions by maximising the smallest overlap, here incidentally realised by $s = 4$ ($s = 5$), among all twenty species.

We employed two stochastic optimisers to obtain the fits to $\mathbf{n}^{\text{ref}}$, a particle swarm optimisation (PSO) and a genetic algorithm (GA). Both can optimise non-convex functions with many local optima in high-dimensional spaces, but we found our implementation of PSO to yield better results more quickly than the GA that we had adapted from the openGA library, see Supplementary Ref. [4]. We optimised in the initially large $(17 \times S)$-dimensional ($(14 \times S)$-dimensional in

the noninteracting case) search space by (i) expanding and shrinking individual dimensions adaptively and (ii) re-initialising the search in 20 subsequent runs each inheriting the adapted search space of the previous run. Through testing on few-species subsystems of the BCI data, we observed that this procedure provides a satisfactory trade-off between computing time and fit quality. Fixing all parameters except the three interaction parameters for each species to their fitted values, and further optimising in the then much reduced parameter space, we only found marginally better fits to the density data, which suggests that the optimisation had already converged to a good local optimum.

Notes on DFTe-based dynamics across temporal scales. Aiming at the simultaneous integration of ecosystem drivers on all time scales, we propose to extract the ecosystem dynamics from trajectories in the space of DFTe energies. Inspired by the mathematical structure of DFTe, we identify two promising routes for addressing time evolution on the (short) dispersal and the (intermediate) ecological time scale, respectively: We propose that processes on the dispersal time scale enter our formalism at the level of the selfconsistent equations of DPFT, specifically through an alignment energy (akin to dipolar interactions of quantum gases) that iteratively steer the directed flow of nonequilibrium density distributions into the states of lowest energy for fixed abundances. Encouraged by our results on Veilleux's predator–prey system in Fig. 5, we also hypothesise that the set $\mathcal{H}(\mathbf{N})$ of these states, spanned by all possible abundances $\mathbf{N}$, is the platform on which time-dependent processes on the ecological time scale evolve through Newtonian-type dynamics.

The simultaneous temporal drivers of ecosystem dynamics are drift (stochasticity of densities and abundances), speciation (emergence of new species), environmental changes, and time-dependent resources—in addition to temporal properties of species, such as situation-specific dispersal through space, growth rates, and time lags in response to stimuli [4], see also Supplementary Ref. [5]. These drivers exert their influence on different time scales, which overlap depending on the involved processes and species. For example, speciation accompanies the evolutionary time scale, which is often tagged to long-term geological processes and climate. Drift happens at shorter time scales, such as those associated with the rate at which individuals disperse through space, but its accumulated effects can influence long-term features like extinction rates. The intermediate ecological time scale is characterised by population changes induced by reproductive cycles, growth and predation rates, and accompanies short-term environmental variability such as seasons, weather, cycles of day and night, or tides. See Supplementary Ref. [6] for a review on transient phenomena in ecology that stretch across these time scales.

We propose to capture this overlapping trinity of time scales by simultaneously augmenting the DFTe framework with three types of time evolution. The according original hypotheses and mechanisms proposed below are a road map for creating a universal tool that predicts the nonequilibrium dynamics of complex ecosystems across all scales of time and space.

To establish a universally suitable starting point for ecosystem dynamics, we propose to identify datasets that suggest adiabatic evolution (a succession of time-independent equilibria for slowly changing external parameters), which drags the system along with the evolving equilibrium. We can then model the 'external dynamics' of such time-(t-)dependent environments $\mathbf{V}^{\text{env}}(t)$ and resources $\boldsymbol{\rho}(t)$ as extensions of their static versions by fitting and predicting density data in the fashion of Figs. 2–6. For example, the measured response of a species s to a climatic change yields a parameterised $V_s^{\text{env}}(t)$ for modelling natural settings—much like the quadratic environment in equation (24) is informed by the species' static response to a temperature gradient in a simple setup and used in a complex setup. These t-dependencies (potentially, on all time scales) promote the global minimum $\mathcal{H}(\hat{\mathbf{N}})$ of E to a time-dependent attractor $\mathcal{H}(\hat{\mathbf{N}}, t)$. First targets should be systems with negligible speciation and drift. Both mechanisms can eventually enter the time-dependent space of DFTe energies $E[\mathbf{n}, \boldsymbol{\mu}](\mathbf{N}, t)$ (of which $\mathcal{H}(\mathbf{N}, t)$ is one slice and $\mathcal{H}(\hat{\mathbf{N}}, t)$ one point) via explicit t-dependent recipes at the level of external dynamics and/or the DPFT loop of equation (21). Note that speciation and extinction manifest in a time-dependent dimensionality of $\mathcal{H}(\mathbf{N}, t)$. This agenda will either render the existing functional structure of E sufficient or identify refined forms of its components.

The time dependence of densities and abundances arise through external dynamics on all time scales, superimposed with species-specific 'internal dynamics' on the dispersal and ecological time scale. We propose to capture the internal dynamics with two internal time-evolution mechanisms. One is induced by the selfconsistent DPFT loop of equation (21) itself, which iteratively transforms nonequilibrium density distributions $\mathbf{n}$ into the state of lowest energy for given abundances $\mathbf{N}$. This form of time evolution implicitly contains a dissipative mechanism that permits the existence of a trajectory towards the DFTe hypersurface $\mathcal{H}(\mathbf{N}, t)$. Suppose, for example, that a drift event removes one out of N individuals. The formerly equilibrated density distribution is now out of equilibrium and will approach the new attractor $\mathcal{H}(N - 1)$ with a rate that is determined by time-dependent species-specific admixing parameters $\theta_s(t)$, see equation (21). Their t-dependence

encodes how well each species copes with an altered (effective) environment and has to be informed by real data. We mean this 'θ-equilibration' to exert its influence primarily on the dispersal time scale, where a fixed abundance is redistributed due to (and in proportion to the magnitude of) short-term disturbances. This includes the scenario of a perpetually changing $\mathcal{H}$, for example due to periodic driving, which can prohibit the system from ever θ-equilibrating. Inspired by the formalism of fluid dynamics, we may establish a velocity field $\mathbf{v}(\mathbf{r}, \boldsymbol{\theta}) = \{v_1(\mathbf{r}, \theta_1(t)), \ldots, v_S(\mathbf{r}, \theta_S(t))\}$ that connects successive density distributions. Such a velocity field describes the dissipative flow of the density distribution towards its lowest-energy configuration on $\mathcal{H}$ and can itself put constraints on the DFTe energy functional: We hypothesise that this back reaction supplements the (either static or explicitly t-dependent) DFTe energy with a velocity-dependent component $E_{\mathbf{v}}$, which vanishes at θ-equilibrium, where $|\mathbf{v}| = 0$ everywhere, but which favours specific velocity alignments during θ-equilibration. Such a dynamic energy component selfconsistently steers the flow of densities by balancing the cost of 'misaligned' velocity vectors (inspired, for example, by dipole-dipole interacting quantum gases) against all other energy components. This agenda aims at predicting population redistributions on the dispersal time scale, such as the flow of migrating wildebeests, swarming locusts, or schools of fish, see Supplementary Fig. 16. Related velocity fields of microswimmers in a hydrodynamic setting have been studied, see Supplementary Ref. [7].

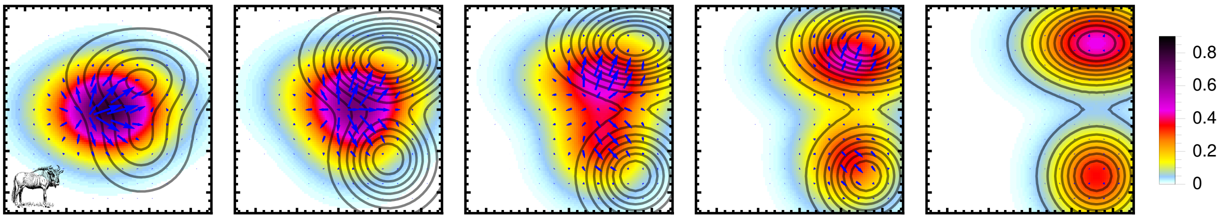

Supplementary Fig. 16. **Hypothetical migration of a wildebeest population.** The five panels illustrate (snapshots of) the velocity field $\mathbf{v}$ (blue arrows), which (i) is associated with the flow of a hypothetical density distribution of wildebeests (colour-coded), (ii) is induced by a time-dependent environment (precipitation, contour lines), and (iii) accompanies the θ-equilibration of the density distribution (towards the right panel).

We propose to supplement the explicit external dynamics and the θ-equilibration with a third mechanism of time evolution that operates predominantly on the ecological time scale. Motivated by our predator–prey results presented in Fig. 5, we argue that species-specific growth rates constrain the possible trajectories that a system can take on $\mathcal{H}(\mathbf{N})$. In particular, a steepest de-

scent along $-\nabla_{\mathbf{N}}\mathcal{H}(\mathbf{N})$ towards $\mathcal{H}(\hat{\mathbf{N}})$ or even the approach of $\mathcal{H}(\hat{\mathbf{N}})$ along any trajectory may be forbidden. The latter scenario entails an 'excitation' energy ΔE above $\mathcal{H}(\hat{\mathbf{N}})$, which cannot be dissipated. Then, ΔE implies steady-state cycles and terminal trajectories in predator–prey systems akin to those hinted at in Fig. 5, or oscillating abundances due to time lags between resource consumption and growth. In Fig. 5, we observe abundance changes over intermediate time scales due to birth and death in an environment that changes slowly enough for $\mathcal{H}(\mathbf{N})$ to be considered static. Under the assumption that the dispersal time scale is much shorter than the ecological time scale, the density distributions θ-equilibrate to $\tilde{\mathbf{n}}$ before significant changes in $\mathbf{N}$ occur, such that we trace a trajectory on $\mathcal{H}(\mathbf{N})$. Of course, in reality the events and mechanisms of the different time scales are at work simultaneously. We may single out the ecological time scale only if it is well separated from the evolutionary time scale and if short-term variations at the dispersal time scale are negligible. Indeed, these conditions are met by Veilleux's predator–prey steady states, which evolve in a uniform time-independent environment void of evolutionary events.

We suggest to extract the relationship between generational rates (e.g., birth or predation rates) and ΔE by matching time-series data with suitable equipotential lines $\mathcal{H}(\hat{\mathbf{N}}) + \Delta E$ on a static $\mathcal{H}(\mathbf{N})$. Generally, t-dependent excitations $\Delta E(t)$ accompany a t-dependent $\mathcal{H}$ that is, for instance, driven by t-dependent environments. Analogous to particles in Newtonian mechanics, we can propagate the abundances $\mathbf{N}$ through the recipe

$$\left\{\begin{array}{rcl} \mathbf{N} & \mapsto & \mathbf{N} + \Delta t\,\partial_t\mathbf{N} \\ \partial_t\mathbf{N} & \mapsto & \partial_t\mathbf{N} - (\Delta t/I)\,\nabla_{\mathbf{N}}\mathcal{H}(\mathbf{N},t) \end{array}\right\}, \tag{32}$$

which allows us to extrapolate limited time series data. Given an energy functional $E[\mathbf{n},\boldsymbol{\mu}](\mathbf{N},t)$, the sole fit parameter in the '$\mathbf{N}$-evolution' of equation (32) is I, which encodes an inertia of the system akin to mass. Discrepancies between $\mathbf{N}$-evolution and experimental data will help to refine E and, consequentially, the driving forces induced by the slope of $\mathcal{H}(\mathbf{N},t)$, towards a universal energy functional that endows the geometry of $\mathcal{H}(\mathbf{N},t)$ with predictive power for time evolution on the ecological time scale.

Notes on image sources. The following images are in the public domain:

• The photos in panels b, d, and e of Fig. 1:

https://commons.wikimedia.org/wiki/File:Drosophila-melanogaster-Nauener-Stadtwald-03-VII-2007-10.jpg

https://commons.wikimedia.org/wiki/File:Poa_pratensis_-_Flickr_-_aspidoscelis_(1).jpg

https://plants.sc.egov.usda.gov/ImageLibrary/large/hoju_002_lvp.jpg

(Credit: USDA-NRCS PLANTS Database, USA)

https://plants.sc.egov.usda.gov/ImageLibrary/large/puai_002_lvp.jpg

(Credit: USDA-NRCS PLANTS Database, USA)

doi:10.7295/W9CIL22782

(Photograph by G. Antipa; http://www.cellimagelibrary.org/)

• The BCI map in Fig. 1g:

https://nsf.gov/news/mmg/media/images/map4.jpg (Credit: Smithsonian Tropical Research Institute, USA)

• The silhouettes in Fig. 1f, Fig. 6, and Supplementary Fig. 16:

https://publicdomainvectors.org/en/free-clipart/Red-mushroom-with-dots/76883.html

https://publicdomainvectors.org/en/free-clipart/Bunch-of-grass-vector-illustration/25652.html

https://publicdomainvectors.org/en/free-clipart/Tree-silhouette-vector-graphics/7129.html

https://publicdomainvectors.org/en/free-clipart/Buck/42510.html

https://publicdomainvectors.org/en/free-clipart/Wild-boar-silhouette/55391.html

https://publicdomainvectors.org/en/free-clipart/Vector-illustration-of-snail/30685.html

https://publicdomainvectors.org/en/free-clipart/Leopard-vector-graphics/35615.html

https://www.kisscc0.com/clipart/wildebeest-drawing-line-art-cartoon-public-domain-umvghc/

Supplementary References

[1] Chen, Y. *et al.* Forecasting the dynamics of segregated population distributions at the neighborhood scale using Density-Functional Fluctuation Theory. Preprint at http://arxiv.org/abs/2008.09663 (2020).

[2] Burchill, C. & Kenkel, N. Vegetation-environment relationships of an inland boreal salt pan. *Can. J. Bot.* **69,** 722 (1991).

[3] Trappe, M.-I., Grochowski, P. T., Hue, J. H., Karpiuk, T. & Rzążewski, K. Phase transitions of repulsive two-component Fermi gases in two dimensions. *New J. Phys.* **23,** 103042 (2021).

[4] Mohammadi, A., Asadi, H., Mohamed, S. M. K., Nelson, K., & Nahavandi, S. OpenGA, a C++ Genetic Algorithm Library. *IEEE International Conference on Systems, Man, and Cybernetics*, 2051 (2017).

[5] Vellend, M. Conceptual Synthesis in Community Ecology. *Q. Rev. Biol.* **85,** 183 (2010).

[6] Hastings, A. *et al.* Transient phenomena in ecology. *Science* **361** (2018).

[7] Menzel, A. M., Saha, A., Hoell, C. & Löwen, H. Dynamical density functional theory for microswimmers. *J. Chem. Phys.* **144,** 024115 (2016).
