# Supplementary material for: A density functional theory for ecology across scales
Source: Nat Commun. 2023 Feb 25;14:1089. doi: 10.1038/s41467-023-36628-4 (PMC9968302; doi:10.1038/s41467-023-36628-4)
Supplement: Supplementary file 3 — Reporting Summary [file 41467_2023_36628_MOESM3_ESM.pdf]

nature portfolio

Corresponding author(s): Martin-Isbjoern Trappe

Last updated by author(s): Jan 30, 2023

Reporting Summary

Nature Portfolio wishes to improve the reproducibility of the work that we publish. This form provides structure for consistency and transparency in reporting. For further information on Nature Portfolio policies, see our Editorial Policies and the Editorial Policy Checklist.

Statistics

For all statistical analyses, confirm that the following items are present in the figure legend, table legend, main text, or Methods section.

n/a	Confirmed	
☐	☒	The exact sample size (n) for each experimental group/condition, given as a discrete number and unit of measurement
☒	☐	A statement on whether measurements were taken from distinct samples or whether the same sample was measured repeatedly
☒	☐	The statistical test(s) used AND whether they are one- or two-sided<br>*Only common tests should be described solely by name; describe more complex techniques in the Methods section.*
☒	☐	A description of all covariates tested
☒	☐	A description of any assumptions or corrections, such as tests of normality and adjustment for multiple comparisons
☐	☒	A full description of the statistical parameters including central tendency (e.g. means) or other basic estimates (e.g. regression coefficient) AND variation (e.g. standard deviation) or associated estimates of uncertainty (e.g. confidence intervals)
☒	☐	For null hypothesis testing, the test statistic (e.g. F, t, r) with confidence intervals, effect sizes, degrees of freedom and P value noted<br>*Give P values as exact values whenever suitable.*
☒	☐	For Bayesian analysis, information on the choice of priors and Markov chain Monte Carlo settings
☒	☐	For hierarchical and complex designs, identification of the appropriate level for tests and full reporting of outcomes
☒	☐	Estimates of effect sizes (e.g. Cohen's d, Pearson's r), indicating how they were calculated

Our web collection on statistics for biologists contains articles on many of the points above.

Software and code

Policy information about availability of computer code

Data collection	No software was used for data collection. We did not collect any original data for this research.
Data analysis	All the code used in this study is available in open-access repositories. The DFTe formalism for the heterogeneous systems is part of the C++ software package 'mpDPFT', available at https://doi.org/10.5281/zenodo.6999439. Analyses for the case studies of Figs. 2–7 are available as Mathematica notebooks at https://doi.org/10.5281/zenodo.7002198.

For manuscripts utilizing custom algorithms or software that are central to the research but not yet described in published literature, software must be made available to editors and reviewers. We strongly encourage code deposition in a community repository (e.g. GitHub). See the Nature Portfolio guidelines for submitting code & software for further information.

Data

Policy information about availability of data

All manuscripts must include a data availability statement. This statement should provide the following information, where applicable:

- Accession codes, unique identifiers, or web links for publicly available datasets
- A description of any restrictions on data availability
- For clinical datasets or third party data, please ensure that the statement adheres to our policy

The experimental data used in this manuscript were extracted from Refs. [1, 25, 34, 42, 54]. The fruit-fly data from Ref. [25] area available at https://doi.org/10.1038/s41467-018-05750-z. We digitised Fig. 6 of Ref. [1], available at https://doi.org/10.2307/1937747, for displaying the experimental data on competing algal species in Extended Data Tab. 2. For our case study on three grasses, we obtained the above-ground biomass data in monoculture and mixture by

digitising Fig. 3 of Ref. [34], available at https://doi.org/10.1139/b91-310. For our predator–prey case study we used the digitised data of Ref. [36], which is provided in the appendix of Ref. [54], available at https://doi.org/10.1098/rspb.2000.1186. The data for Barro Colorado Island are accessible through Dryad at https://doi.org/10.15146/5xcp-0d46.

Human research participants

Policy information about studies involving human research participants and Sex and Gender in Research.

Reporting on sex and gender	n/a
Population characteristics	n/a
Recruitment	n/a
Ethics oversight	n/a

Note that full information on the approval of the study protocol must also be provided in the manuscript.

Field-specific reporting

Please select the one below that is the best fit for your research. If you are not sure, read the appropriate sections before making your selection.

☐ Life sciences ☐ Behavioural & social sciences ☒ Ecological, evolutionary & environmental sciences

For a reference copy of the document with all sections, see nature.com/documents/nr-reporting-summary-flat.pdf

Ecological, evolutionary & environmental sciences study design

All studies must disclose on these points even when the disclosure is negative.

Study description	The study is purely theoretical and uses only data from existing published studies. We developed a novel framework, called density functional theory for ecology, for analysing ecological systems across scales. We applied the framework to six case studies.
Research sample	We chose six case studies that had data appropriate for our novel modelling approach. Five of these were from published papers; one was based on our own synthetic data. In the five published case studies, the samples were completely determined by the original authors. In the first case study, the research sample involved fruit fly densities in heated chambers. In the second case study, the research sample involved algal densities in microcosms. In the third case study, the research sample involved grass abundances in experimental plots. In the fourth case study, the research sample involved abundances of predator and prey microbial species. The fifth case study was the synthetic model. In the sixth case study, the research sample involved tree species abundances in a forest plot.
Sampling strategy	The sample sizes were entirely determined by the original authors of our case studies and thus were not within our control.
Data collection	We did not collect any original data.
Timing and spatial scale	We did not collect any original data.
Data exclusions	We did not exclude any data.
Reproducibility	We did not conduct any experiments.
Randomization	We did not perform any randomisation because we did not conduct any experiments.
Blinding	We did not perform any blinding because we did not conduct any experiments.

Did the study involve field work? ☐ Yes ☒ No

Reporting for specific materials, systems and methods

We require information from authors about some types of materials, experimental systems and methods used in many studies. Here, indicate whether each material, system or method listed is relevant to your study. If you are not sure if a list item applies to your research, read the appropriate section before selecting a response.

Materials & experimental systems

n/a	Involved in the study
☒	☐ Antibodies
☒	☐ Eukaryotic cell lines
☒	☐ Palaeontology and archaeology
☒	☐ Animals and other organisms
☒	☐ Clinical data
☒	☐ Dual use research of concern

Methods

n/a	Involved in the study
☒	☐ ChIP-seq
☒	☐ Flow cytometry
☒	☐ MRI-based neuroimaging
